# Published and unpublished evidence in coverage decision-making for pharmaceuticals in Europe: existing approaches and way forward

**DOI:** 10.1186/s12961-016-0080-9

**Published:** 2016-01-26

**Authors:** Dimitra Panteli, Alexandra Nolting, Helene Eckhardt, Michael Kulig, Reinhard Busse

**Affiliations:** Department of Health Care Management, Berlin University of Technology, Berlin, Germany; Federal Joint Committee (G-BA), Berlin, Germany

## Abstract

**Background:**

Dissemination bias occurs when only some results emerging from clinical research reach their intended audience in the knowledge translation process. Given that coverage decisions increasingly rely on evidence, it is important to explore the types of evidence considered. This paper aimed to examine the evidence base used by regulatory institutions involved in pricing and reimbursement of pharmaceuticals in a broad range of European countries, as well as their awareness of and approach towards dissemination bias.

**Methods:**

A mixed methods approach was adopted. Regulatory documents and published literature were identified in systematic searches and relevant documents were analysed. An online survey was carried out to verify and expand insights.

**Results:**

Forty-two relevant regulatory documents and 10 publications were included. The survey had a 35% response rate, yielding valid responses for 13 countries. A fragmented impression was obtained for most countries indicating a general lack of transparency regarding both processes of decision-making and approaches towards unpublished information. Dissemination bias was rarely consistently considered. Practices for the identification and inclusion of all available evidence varied considerably, as did the influence of missing evidence on decision-making. Differences were often attributable to the regulatory context and/or institutional principles.

**Conclusions:**

Best practice is difficult to generalize given the identified variations. Individual exemplary practices support the necessity for institutional exchange at international level. Increased institutional commitment to transparency of methods and processes should be advocated.

**Electronic supplementary material:**

The online version of this article (doi:10.1186/s12961-016-0080-9) contains supplementary material, which is available to authorized users.

## Background

Dissemination bias in the knowledge translation process occurs when “*the dissemination profile of a study’s results depends on the direction or strength of its findings*” [1]. It can take different forms, most notably that of publication bias (studies with positive, statistically significant results are favoured for publication) or selective reporting/outcome reporting bias (for a given study, favourable outcomes with significant effects are more likely to be reported) [2]. In general, if only positive and significant results are published, dissemination bias can lead to an overestimation of positive effects and an underestimation of both lack of effect and, most importantly, harm.

Thus, the partial disclosure of findings can have substantial impact on patient safety and quality of care. Furthermore, given that decisions related to coverage and other elements of health policy are increasingly relying on evidence, missing information can also negatively impact areas such as resource allocation. Finally, the partial or non-disclosure of human trials is also problematic at an ethical level. Study participants assume the risks inherent in any experiment, at least partially with the intent of advancing societal good (in this case the generation of new knowledge that will help future patients). If trial results do not flow into knowledge translation, study participants have exposed themselves to harm without any benefit [3, 4].

The extent of the phenomenon, as well as its potential effects, has been the focus of an increasing volume of scientific research (indicatively [2, 5–10]). In light of a number of initiatives towards more comprehensive and representative evidence availability by national and international institutions, as well as the scientific community and industry, the European Commission funded comprehensive research on understanding how dissemination bias impacts different areas of the knowledge generation and translation process in healthcare-related decision-making and what strategies are and could be applied to address it.

In this respect, one of the aims of the 7th Framework Programme-funded project “OPEN” was to identify practices related to publication bias on behalf of the “*main parties involved in approving, funding, conducting, publishing, and disseminating clinical research*” [11]. Part of the research carried out within the OPEN project investigated dissemination bias in the context of coverage decisions for pharmaceuticals. Work Package 10 examined pricing and reimbursement processes in 36 European countries, focusing on the role of regulatory institutions responsible for pharmaceutical coverage, the evidence base they use for their decisions and their awareness of and approach towards dissemination bias. This article presents findings from research carried out to address the latter two points.

## Methods

### Rationale

The aim of this research was to capture practices in a broad range of European countries (n = 36), including EU Member States, candidate and accession countries as well as the European Free Trade Association. The practices of active regulatory institutions charged with the assessment of and/or decision-making on the value of pharmaceuticals for public reimbursement or pricing at the national level were explored. Only institutions in the aforementioned countries were eligible. Processes at regional level were not specifically targeted in countries with decentralized systems, with the exception of the United Kingdom, where the systems for England and Wales, and Scotland were studied separately.

The goal was to compile as broad a range of relevant information as possible on a number of areas related to the consideration of unpublished or incomplete data by regulatory bodies responsible for coverage decisions in European countries (Additional file 1: Table S1). A mixed methods approach was adopted including (1) document analysis informed by desk research and (2) a survey of institution representatives.

### Document analysis

The document analysis on institutional policies towards publication bias included (1) a systematic search for regulatory documents on the websites of all included institutions; (2) a systematic literature search in three databases; (3) the selection of relevant material from both searches; (4) the extraction of information from the final document pool; and (5) the synthesis of information and identification of evidence gaps.

Regulatory documents (guidelines, manuals, directives, submission requirements, etc.) were searched for in a systematic manner using both the site map and search function of each website to identify online information and linked material. In parallel, literature searches were run in PubMed, EMBASE and the Cochrane Library (see Additional file 2 for search strategies). Inclusion criteria for the selection of both regulatory documents and publications were specified in advance (Additional file 3: Table S2). The reference lists of included publications were searched to identify eligible material not captured by the initial search. Both regulatory documents and publications were identified in April 2012. Two independent reviewers were involved in all steps of the systematic process and discrepancies in screening documents and search hits for relevance were solved by discussion and consensus. Information was extracted from all documents using a structured extraction sheet based on the elements in Additional file 1: Table S1. Google Translate was used for documents that were in languages other than English, French, German, Russian and Swedish, and interpretations were checked by two researchers separately.

### Survey of relevant institutions

To supplement the findings from the document analysis and to ensure the completeness and validity of information, a descriptive online survey was conducted among included institutions. The survey was based on a structured questionnaire which was designed with the purpose of obtaining comparable responses while minimizing effort for participants (see Additional file 4 for the full survey tool). The content and structure of the survey questions were developed by two researchers and reviewed by another two, designed to correspond to the thematic foci used to guide the document analysis. Appropriate contact persons for each institution were identified by means of author networks and online resources while additional contacts were sought at a later point due to low response rates. The survey was uploaded to Surveymonkey™ in August 2012 and email invitations were sent out. Up to five periodic reminders were sent to non-respondents until November 2012.

### Result synthesis and presentation

Results from the document analysis and survey are presented in a synthesized manner, organized by thematic focus. Country-specific information stemming from the document analyses is followed by the country name and a reference to the corresponding document, while for survey insights only the country name is provided.

## Results

### Information yielded by the searches and survey response rate

**Fig. 1:**
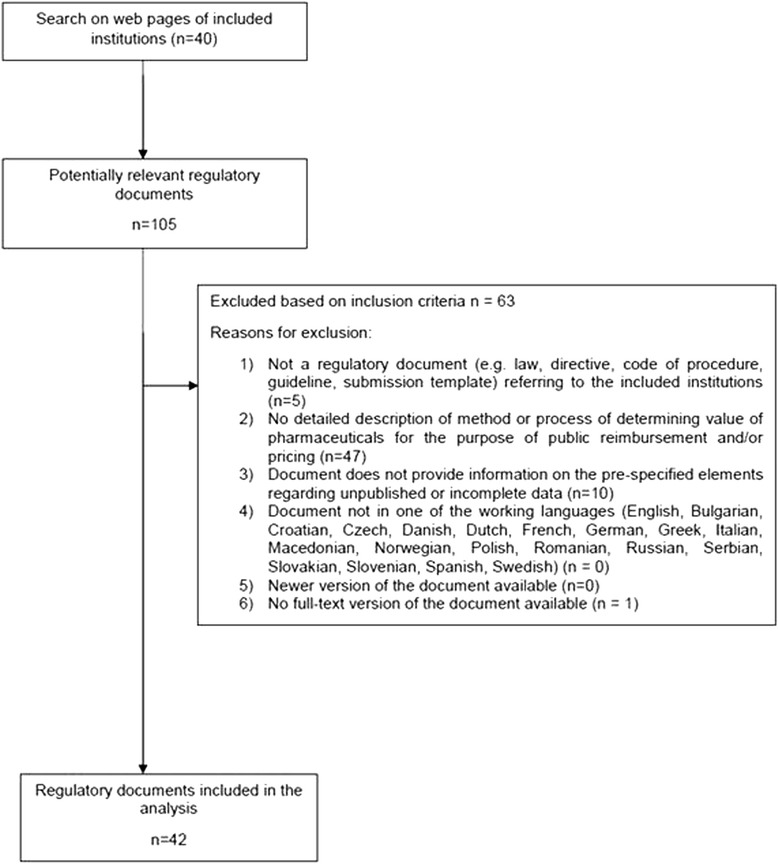
Selection of regulatory documents

**Fig. 2:**
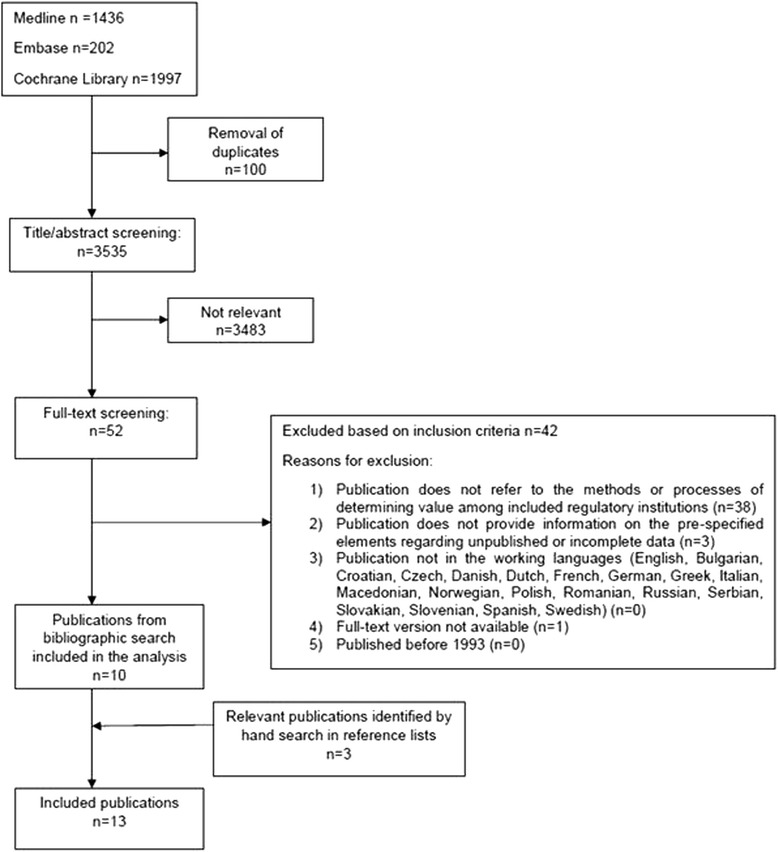
Selection of publications

Overall, 105 potentially relevant regulatory documents were identified and screened, upon which 42 were selected for the analysis (see Fig. 1 for selection flowchart and reasons for exclusion). The systematic database search retrieved 3,535 citations after the removal of duplicates. Of those, 3,483 citations were excluded following title and abstract screening based on the pre-specified inclusion criteria. Out of 52 remaining citations checked for relevance in full text, 10 publications were deemed eligible for analysis; their reference lists yielded three additional relevant publications (see Fig. 2 for selection flowchart and reasons for exclusion). An overview of information availability per country is provided in (Additional file 1: Table S1). A list of all included regulatory documents and publications is available in Additional file 5.

Despite repeated reminders sent to institutional contacts for the survey, information was not forthcoming in several cases (n = 20). Two institutions replied that they would not be participating in the project due to time constraints. In a further two cases only the first question of the survey was filled out with the indication of lacking knowledge on subsequent items. One of the respondents included in the analysis only filled out the first two parts of the questionnaire. Overall, 13 valid responses could be processed corresponding to an approximate 35% of the 37 institutions initially contacted.

The combined efforts described above yielded information on 26 of the 36 countries included in the initial pool. However, the extent of information varied considerably across countries: while a comprehensive idea could be gained for some, such as England and Germany, only partial aspects of the topics examined were covered for others (see in Additional file 1: Table S1 for information availability per country). Thus, the following sections do not provide a full information profile for each country. Quantifying statements (‘most’, ‘the majority’, etc.) refer to the totality of countries for which information was available on each topic. Fraction denominators refer to valid responses per survey question.

### Information retrieval and identification of unpublished data

#### General approach to evidence for coverage decisions and submission requirements

Several institutions mentioned the methods of evidence-based medicine as pillars that should underpin assessment processes (Austria [12]; Slovakia [13]; Poland [14]; Germany [15]). All institutions participating in the survey (13/13) declared that they attempt to identify all relevant scientific evidence for their assessments, whether they perform research themselves or rely on submissions. However, non-systematic approaches are either usually or occasionally adopted in five countries when research is performed within the institution (Austria, Czech Republic, Iceland, Serbia, Sweden).

Specifications on what should be included in applications in countries where the assessment is primarily based on manufacturer submissions vary in form and detail (Austria [16]; France [17]; Germany [15]; England & Wales [18]; Scotland [19]; Sweden [20]). A full list of relevant studies and definitely all studies that were used for product authorization (licensing) are required in most cases, while in both Germany [9, 15] and Scotland [19] study reports and protocols should be included. Unpublished studies are required in some cases (Austria, Germany, France, Portugal) along with ongoing trials in France and Germany. In Luxembourg, license holders have to provide supporting evidence in the form of at least one study when applying for reimbursement [21], while in Switzerland the most important studies need to be included [22].

An overview of sources used during evaluation among respondents to the survey can be found in Additional file 6: Table S3.

### Sources and types of published information

Submission requirements for manufacturers and institutional method guides both specify the sources from which information should be drawn. A minimum of two databases is usually required for a search to be considered systematic, while it is not always strictly specified which two need to be chosen. The databases most frequently mentioned can be found in Additional file 7: Table S4. The Institute for Quality and Efficiency in Health Care (IQWiG) in Germany [23] and the Agency for Health Technology Assessment (AOTM) in Poland [14] explicitly mention that systematic searches need to aim for the highest possible sensitivity in order to ensure that no relevant studies are missed.

Regarding limitations to the representativity and comprehensiveness of the evidence base, language of publications (Germany [23], Ireland [24], England & Wales [18]) and timeline of the search (Germany [25], England & Wales [18]) are recognized as significant aspects that need to be taken into account and documented both in cases of submissions and institutional research. While a number of countries (4/13) do not enforce a language restriction for evidence retrieval, some only include publications in English (Czech Republic, Malta, Serbia, England & Wales) or in English and the country’s own language (Austria, France, Portugal, Sweden). Iceland also includes publications in other Nordic languages.

When institutions involved in evidence assessment perform additional research, different approaches exist with regard to the types of publications drawn upon for assessment. Some institutions prioritise the utilisation of secondary publications such as health technology assessment (HTA) reports or systematic reviews and revert to the search for primary literature if newer information is available or the evidence base provided in the secondary sources is not deemed adequately robust (Denmark [26]; Germany [23]; Poland [14]).

### Approach towards unpublished information

While all (13/13) respondents to the survey mentioned that their institutions take published data into account for coverage decisions (Additional file 6: Table S3), only seven (Czech Republic, Germany, Finland, France, Iceland, Poland, Sweden) explicitly stated that unpublished information is also considered. Furthermore, the extent to which additional effort is invested in identifying unpublished evidence during evidence procurement varies. The National Institute for Health and Care Excellence (NICE) in England made it necessary to include all published and unpublished information in assessments in 2004 [27, 28]. The Irish Health Information and Quality Authority also explicitly states that all attempts should be made to identify relevant information beyond published data [29] and the German institutions insist on an evidence base as comprehensive as possible, be it in submissions from the industry or in their own research [15, 23]. Interestingly, while assessments performed by the Association of Social Security Institutions in Austria should attempt to capture all evidence [12], applicants wishing to include their products in the reimbursement list (*Erstattungskodex*) can limit their systematic evidence submission to published literature [16].

As can be seen in Additional file 6: Table S3, several methods are employed in order to identify unpublished information. Indicatively, these include searches in trial registries, most commonly the EU Clinical Trials Register (5/8 responses) and ClinicalTrials.gov (4/8 responses), requests to the industry and/or publication authors (Germany [15, 23], Croatia [30], Poland [14], Turkey [31], England & Wales [32]), and the active involvement of stakeholders such as health professionals and manufacturers (England & Wales [27, 32–34]). In Iceland, patient representatives are also consulted. The search for and utilisation of grey literature is not uniformly adopted. For instance, while in some cases conference abstracts are sought out as sources of information, other institutions specify that they are not acceptable as evidence for assessment (The Netherlands [35], Scotland [19]).

The Haute Autorité de Santé (HAS) in France actively seeks out the opinion of equivalent institutions in other countries to ensure evidence completeness [17] and the Association of Social Security Institutions in Austria mentions the utilisation of international networks [16]. Overall, the exchange of information among institutions determining the value of pharmaceuticals for pricing and reimbursement purposes in the European context seems to be taking place on a case-by-case rather than a routine basis. National, European and international medicines agencies are either routinely or occasionally consulted by all institutions who responded to the survey. Information from marketing authorization agencies required for assessment usually takes the form of public assessment reports at national or European level (European Public Assessment Report; 8/10 responses), while the opinion of the Committee for Medicinal Products for Human Use is just as often taken into account (8/10 responses). Clinical study reports of marketing authorisation studies are also commonly used (6/10 responses), while study protocols are required and included in Germany and France. Periodic safety update reports are routinely included in Finland, France, Slovenia and the United Kingdom while other approaches (EudraVigilance, Food and Drug Administration Medical Reviews or Adverse Event Reporting System) are also employed by individual institutions.

### Evaluation of evidence quality and assessment of risk of bias

Information on the assessment of evidence quality ranged from general approaches towards quality control to more specific methodological issues and concrete measures for checking evidence for a range of biases.

General approaches toward evidence quality included issues such as the awareness that bias due to poor quality of studies should be taken into consideration (Ireland [29]) or internal and external quality checks (Austria [12]). More specifically, while validated reporting standards such as CONSORT and STROBE are used in France, Germany and Portugal, checklists developed particularly for the Norwegian context are to be used for the same purpose in Norway [36]. These include evidence hierarchies, which are also used in Austria, Croatia and Germany. In Germany, both the Federal Joint Committee (G-BA) and the IQWiG have clear guidelines on the categorization of risk of bias for individual studies, while the latter excludes studies with high risk of bias from its own analyses.

Specific measures related to evidence quality and potential biases identified during the document analysis include an explicit preference for peer-reviewed publications for modelling in pharmacoeconomic research (The Netherlands [37]), standards for the comparability of studies that can be included in indirect comparisons and the validity of surrogate outcomes (Norway [36]), and checking international evidence for transferability (Croatia [30], Norway [36]).

### Specific methods for identification, analysis and presentation of unpublished information

The consistency of retrieved information is ascertained both within the same document (8/12 responses) and across information sources (8/12 responses). If inconsistencies exist, the preferred source of information are clinical study reports in Germany, France and Slovenia, and manufacturer submissions in the United Kingdom, while most institutions (6/12 responses) prioritise published articles. Five respondents indicated that selective outcome reporting is controlled before the assessment begins (Austria, Germany, France, Malta, Sweden). Comparison between study protocols and reports/publications (Germany) or the retrieval of the original study material (Malta) were the specific methods mentioned. Only two respondents indicated explicitly checking retrieved evidence for risk of publication bias (Germany, Portugal).

Once identified, most institutions that consider unpublished evidence (5/12 responses) use different validity criteria for published and unpublished studies with the exception of Ireland [24], Finland, France and Germany. With regard to using the data in analyses, some institutions clearly state that missing data in meta-analyses need to be calculated or approximated (Germany [23, 25], France [17], Poland [14], Scotland [19, 38]). Uncertainty is explored by means of sensitivity or sub-group analyses by some (4/12 responses: Germany, France, Portugal, Sweden). France, Germany (IQWiG) and England include ongoing studies in their discussion of results.

### Consideration of unpublished data in the formulation of conclusions and recommendations

The AOTM in Poland explicitly requires assessment of whether the inclusion of only published studies can lead to an incorrect interpretation of the review results due to publication bias, but no specifics are given as to how this should be achieved [14]. In Germany, the IQWiG has a matrix to determine how bias could influence its conclusions and recommendations [23], while the G-BA stipulates that the additional benefit of a given drug is considered as not proven if the submission is incomplete or aspects of methodology or results remain unpublished [15]; it further stipulates that the result of sensitivity analyses based on ‘high’ or ‘low’ risk of bias can considerably influence the institution’s conclusions [25]. Several respondents to the survey (7/12) indicated that the influence of unpublished information is to be explicitly considered when interpreting assessment results.

### Impact of incomplete evidence on decision-making

If submitted or retrieved evidence is judged to be incomplete, different processes are followed in different countries: most commonly, the assessment can conclude that benefit has not been proven (9/12 responses). In other cases, the conclusion can be negative or decision-making overall can be postponed (9/12 responses). The postponement of the assessment process and the complete refusal of reimbursement are also possible (7/12 responses), as is the refusal of high pricing or a negative recommendation regarding the utilization of the pharmaceutical in question (5/12 responses). Lastly, decision-making on reimbursement and assessment can be refused entirely (4/12 responses).

In Germany, for example, the G-BA can block an assessment if the documents submitted by manufacturers are deemed incomplete [25, 39]. Meanwhile, NICE requires manufacturers and sponsors of technologies under multiple technology appraisal to sign a statement declaring that all material relevant to the appraisal has been disclosed. The institute can also terminate a single technology appraisal if there is no or only partial evidence submitted until manufacturers or sponsors declare that they wish to provide a full evidence submission, upon which the appraisal can be reinitiated. Furthermore, the Association of the British Pharmaceutical Industry’s Code of Practice asks that all manufacturers register all their clinical trials according to the Joint Agreement of the International Federation of Pharmaceutical Manufacturers and Associations (from Phase II onwards and on a publicly accessible website) [40].

### Management of confidentiality and transparency

#### Information considered ‘in confidence’

Most (8/12) of the institutions who responded to the survey accept submissions including commercial-in-confidence data. As a rule, it is the owners/manufacturers who ultimately select which data will be labelled ‘in confidence’ when filing a submission, but they still have to fully justify the need for confidentiality.

NICE only accepts submissions including commercial-in-confidence data in exceptional cases after prior authorization and expects that all information on the methodology of a given study be in the public domain. It further stipulates that HTA summaries are to be based on all data and be CONSORT or PRISMA compliant [18, 40]. In Germany, while submissions to the G-BA can include confidential documentation which needs to be marked clearly [39], the IQWiG does not accept commercial-in-confidence data at all, asking submitting manufacturers to sign a confidentiality waiver [41]. In Ireland, a summary of information used in HTA should be publicised even if the underlying data has not been fully disclosed up to that point in order to ensure transparency [24], whereas in the Netherlands final reports should ideally only include public data [35].

NICE is the only institution in the sample to explicitly differentiate between commercial-in-confidence and academic-in-confidence data, assuming that the latter can be discussed during appraisal meetings despite the fact that they need to not be widely publicly disclosed in order not to prejudice the publication of information in scientific form [40].

### Publication of appraisals and results of the decision-making process

**Fig. 3:**
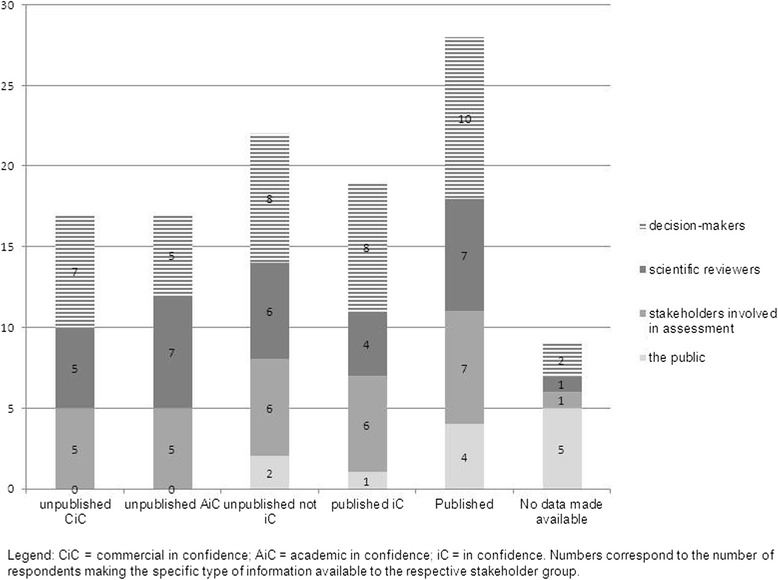
Types of data made available to different types of stakeholders by surveyed institutions. CiC, Commercial in confidence; AiC, Academic in confidence; iC, In confidence. Numbers correspond to the number of respondents making the specific type of information available to the respective stakeholder group

Different approaches to ensure confidentiality while maintaining transparency exist. The activities of the Bulgarian Transparency Committee are announced online and a biannual activity report is published on the Ministry website. In addition, members serving on the Committee are bound by secrecy regarding all information they become aware of during the performance of their duties [42]. In Belgium and France, this secrecy extends to the Committee’s secretariat and external experts [43, 44]. In Germany, while the G-BA will publish the full documentation on the appraisal online unless there are confidentiality issues that need to be observed, the IQWiG makes all information, including stakeholder views, available on its website without exception [23, 39]. The types of information disclosed to different stakeholders among respondents to the survey are shown in Fig. 3.

Also influencing practices in this respect is the issue of freedom of information and its application in healthcare and pharmaceutical appraisal. In the Swedish context, documents kept by public authorities are public. However, both the disclosure and the privacy rule apply to coverage decisions and therefore some information can remain confidential. Nevertheless, it is the goal of the Dental and Pharmaceutical Benefits Agency to design dissemination of its decisions in such a way that they can be fully publicised [20]. A similar situation is also described in England, where the 2000 Freedom of Information Act principally allows anyone to obtain information they request from public authorities and NICE has to consult the owner of in-confidence data to respond to request on a case-by-case basis [40].

### Management of scientific independence and potential conflicts of interest (CoI)

The type of statement describing potential CoI and persons mandated to provide it varies according to the context and process of evaluation in each country. Experts serving as consultants during the assessment or appraisal process are most commonly obliged to report relations (8/12 responses). Members of committees responsible for coverage decisions are uniformly required to report CoIs; these can lead to exclusion from the entire evaluation process (7/12 responses) or a restriction in voting rights (4/12 responses). Members of the Swiss Federal Drug Commission (Eidgenössische Arzneimittelkommision) also need to recuse themselves upon applicant request [22]. The type of action depends on the type and gravity of the conflict in France and Germany, while the declaration itself is enough and no action is taken in Austria.

All individuals participating in appraisal processes carried out at the HAS in France or NICE in England need to declare their CoI on appointment, at advisory Board meetings and for publications. Senior staff and institute employees at both institutions should not have (major) pecuniary CoI [32, 45–47]. Declarations are taken down in meeting minutes and are therefore public. However, as a rule, CoI are not publicised (9/12 responses). Summary statements are available to the public in Germany, Portugal and Sweden.

The HAS implements CoI declaration matrixes which differ for external experts and employees of the institution participating in the appraisal. These are put together by a separate unit within the institution, a group which is also responsible for looking at international best practices on reporting CoI and amending the matrixes accordingly. The declared CoI is taken into consideration before a working group for the appraisal is formed and eligible candidates are short-listed. HAS’s guide cited here provides great detail on what constitutes a major and minor, a direct and indirect CoI, and for whom [45].

## Discussion

Based on the identified institutional approaches, concerns about dissemination bias were not consistently embedded in coverage decision-making processes across countries. Given the differences observed among formal systems for the reimbursement and pricing of pharmaceuticals in Europe, a certain degree of variation regarding institutional practices towards procuring and evaluating evidence for assessment and the consideration of unpublished data was to be expected. It is intuitive to assume that the level of awareness towards dissemination bias and the inclusion of provisions for unpublished data in institutional practices may depend on the maturity and complexity of the formal coverage system itself. However, our results show that even in well-established systems, there are not always explicit considerations on the potential effects of unpublished data. Furthermore, some responses to the survey indicate that questions requiring background knowledge on systematic research, and particularly those regarding specific issues on unpublished or incomplete data, were sometimes skipped or lack of knowledge was indicated. Thus, awareness on the existence and meaning of dissemination bias needs to be increased before specific measures can be taken to address the effects of incomplete evidence in coverage decisions.

Given the varied institutional practices toward evidence-based decision-making it is difficult to extrapolate optimal approaches in an overarching way. This is mainly attributable to the fact that existing practices are dependent not only on the legal context in each health system but also on previous experiences and available resources. It is, however, possible to identify exemplary aspects from the perspective of evidence-based decision-making. For example, in the German system, requirements guiding the submission of documents for pricing and reimbursement processes are not only detailed in nature but also almost exhaustively apply the principles of systematic research. Meanwhile, NICE provides clear and transparent directions for various stakeholders in the health system with regard to their contribution to the decision-making process. Likewise, the HAS approach towards CoI and the declaration of these is detailed and transparent. The subsequent full publication of declarations is a unique practice among countries where information was available and it may not be easily transferrable to other legal contexts.

It is similarly difficult to formulate uniform recommendations on issues related to unpublished data that seem to depend on institutional principles. For example, the acceptance of commercial-in-confidence data ranges from excluded (IQWiG in Germany) to almost not restricted (Austria, Croatia). Similarly, the utilisation of conference abstracts and other grey literature as a supplementary source of information is common practice in some countries and ruled out in others. Great variations seem to exist both in the number of sources used and in the level of transparency of methods.

The consultation of trial registers, which is already part of the process in several countries, offers a first line of insight into unpublished information and should be considered by institutions that do not already employ it as routine practice. It is interesting to note that recent research confirmed a high ratio of unpublished study results even among large clinical trials [3, 4, 48]. In response, WHO issued a Statement on Public Disclosure of Clinical Trial Results, endorsing timely and comprehensive registration [49]. While not all unpublished studies will necessarily have results available on the register [50], a consistency check of the body of evidence submitted or collected can still point towards the level of its completeness and help estimate the extent to which a decision would be taken on a sound basis [10]. The rationale behind such consistency checks would need to allow for different types of pharmaceuticals under evaluation – newly authorized substances, for which few studies are likely to be available, may be more prone to selective outcome reporting bias than publication bias. Thus, a valid consistency check would require a comparison with study protocols or full trial reports. Furthermore, most formal systems for pharmaceutical coverage decisions (initially) rely on manufacturer submissions [51]. Therefore, a mandatory declaration of full disclosure by manufacturers can be an important step towards ensuring that all data is taken into account in the process.

Among other national and international resources, the European Medicines Agency (EMA) is also frequently consulted for information. After its policy to widen access to trial documents was halted by the EU General Court in spring 2013 upon objections from the industry, the Agency issued another draft policy on the proactive publication of trial data [52] along with a step-wise approach for the adoption of the policy to “*address the risk of possible unfair commercial use of data while ensuring proactive and non-selective access*”. Following reactions from the scientific community on the type and extent of proposed data access [53], the Agency revised the policy, which was in its new form adopted in October 2014 and became effective on January 1, 2015. The policy stipulates that the EMA database, including clinical trial reports submitted for marketing authorization of medicines as of January 2015, will be made available to the public in line with the new EU Clinical Trial Regulation No 536/2014. The EMA policy acknowledges that, while commercial in-confidence information will be redacted before reports are made public, clinical data can generally not fall into this category [54].

An interesting aspect coming forth from both the document analysis and the survey is that of institutional exchange and/or cooperation. Institutions with extensive experience are sometimes referenced in guidelines from corresponding institutions in other countries. In addition, further institutions of the same national health system, or institutions responsible for determining value in other countries within and outside Europe as well as international HTA networks are considered as additional sources of information for assessment. The possibility of data sharing between regulatory institutions would therefore facilitate evidence completeness. Taking into consideration the differing levels of experience as well as the multitude of approaches in place regarding publication bias, a more organised collaboration could be advantageous, especially for institutions with limited experience.

Finally, one of the main issues that emerged from this research was the lack of transparency regarding the methods behind evidence-based coverage decisions in many countries. Public availability of regulatory documents as well as information from publications regarding evidence procurement, evaluation and utilisation, and its impact on decision-making, was sparse and unbalanced. This mirrors the general lack of transparency which characterizes formal reimbursement processes and leaves room for improvement-[51]. In their framework for classifying coverage decision-making systems based on evidence, Hutton et al. [55] estimated the proportion of information likely to be publicly available on several elements of the process. Our results more or less confirmed that, for some elements, such as general methods and responsibilities, information is more forthcoming, while substantial gaps remain on other areas such as transparency and accountability.

This research provided part of the evidence base informing a stakeholder workshop on dissemination bias carried out in May 2013 by Cochrane Germany. The workshop aimed to issue consensus-based recommendations for a range of key actors in the knowledge generation and translation process. Next to general principles to guide practice for all actors (including awareness-raising, supporting trial registration and multi-register searches, and endorsing rigorous methodology in scientific evidence synthesis), specific recommendations were provided for each group [56]. Regulatory institutions responsible for determining the value of pharmaceuticals for coverage were thus encouraged to:

1. make their methods and processes of benefit assessment publicly available in order to achieve better transparency and understanding;
2. aim for a higher degree of collaboration between institutions to facilitate the detection of further (unpublished) data and to foster data sharing;
3. use the full evidence base on an intervention for their assessments;
4. (publicly) specify their course of action if they find that the evidence base for an assessment is deemed incomplete (e.g. no adequate proof of benefit based on incomplete data set); and
5. request from legislators the following items which will allow the consideration of all study results (disclosure of full protocols and full clinical study reports):
  - a legal obligation for manufacturers to submit all requested evidence
  - public access to EMA databases
  - public access to protocols and full study reports

In order for these recommendations to reach their full potential and be implemented in a meaningful manner, relevant institutions should also consider specific methodological issues emerging from this work given their remit and resources – adopt an approach that is as systematic as possible (e.g. regarding sources of information, evidence type and language etc. – see also recommendation 3, above); consider the consistent use of specific methods, such as funnel plots, to assess likelihood of bias in the evidence base; consult trial registries and perform consistency checks of submitted or identified evidence with study protocols and/or full study reports; and capture and publish potential conflicts of interest for those involved in the evaluation process.

### Limitations

One of the main concerns of the authors during this project was the considerably limited amount of publicly available information for a number of countries on the procurement and utilization of evidence in general and, more specifically, their formal approach to unpublished data. From a methodological viewpoint, this is regrettable for a variety of reasons; existing exemplary practices may not have been identified while practices illustrated in this article may have been overestimated in the overall context of fragmented information. Furthermore, a distinct lack of information remains for a number of countries. Due to the nature of the survey, the low response rate (13/37 institutions) greatly impacted evidence completeness. Later research on other topics suggests that a shorter questionnaire administered by phone could have been more successful in this respect. The utilization of Google Translate comes with its own limitations as to the validity of information extraction. As data collection for this work was completed in 2012, any changes in institutional practices having come into effect in the meantime were not taken into consideration. Especially in light of newer changes at international level (e.g. EMA policy and WHO endorsement mentioned above), it would be interesting to revisit this issue in due time to map relevant progress.

## Conclusions

Despite the fact that the scientific community has been increasingly aware of dissemination bias and its consequences, our results showed that such concerns were sporadically embedded in coverage decision-making processes. There is a distinct lack of transparency regarding both the processes of evidence-based decision-making themselves and the consideration of unpublished data. While underlying differences make it difficult to extrapolate optimal approaches in an overarching way, steps, such as a requirement for manufacturers to submit all data and the encouragement of actions already taken by actors such as the EMA, can be crucial. Exemplary practices regarding the consideration of unpublished data and ensuring transparency were identified; in this respect, enhanced institutional collaboration could contribute to both awareness raising and knowledge exchange.

## Additional files

Additional file 1: Table S1. Overview of information per institution and domain (and list of alternative institutions contacted). (DOCX 32 kb) Additional file 2:
**Search strategies for publications.** (PDF 203 kb) Additional file 3: Table S2. Inclusion criteria for regulatory documents and publications. (DOCX 18 kb) Additional file 4:
**Survey tool used among participating institutions.** (PDF 369 kb) Additional file 5:
**List of regulatory documents organized per country and list of publications.** (PDF 274 kb) Additional file 6: Table S3. Sources used to retrieve information for the assessment of pharmaceuticals among respondents to the survey (n = 13). (DOCX 16 kb) Additional file 7: Table S4. Bibliographic databases used to identify published information among respondents (n = 13). (DOCX 15 kb)

## Abbreviations

AOTM: Agency for Health Technology Assessment (Poland)
CoI: Conflicts of interest
EMA: European Medicines Agency
G-BA: Federal joint committee (Germany)
HAS: Haute Authorité de Santé (France)
HTA: Health technology assessment
IQWiG: Institute for Quality and Efficiency in Health Care (Germany)
NICE: National Institute for Health and Care Excellence (United Kingdom)
