# Supplementary material for: Published and unpublished evidence in coverage decision-making for pharmaceuticals in Europe: existing approaches and way forward
Source: Health Res Policy Syst. 2016 Jan 26;14:6. doi: 10.1186/s12961-016-0080-9 (PMC4727332; doi:10.1186/s12961-016-0080-9)
Supplement: Additional file 1: Table S1. — Overview of information per institution and domain (and list of alternative institutions contacted). (DOCX 32 kb) [file 12961_2016_80_MOESM1_ESM.docx]

**Table 1 Overview of information per institution and domain**

| **Country** | **Institutions contacted during survey** | **Infor-mation retrieval** | **Evidence quality/**  **risk of bias** | **Publi-cation bias in analysis and synthesis** | **Identifi-cation of publi-cation bias** | **Publi-cation bias in conclusions/ recom-mendations** | **Policies for data comp-leteness** | **Manage-ment of confiden-tiality & transpa-rency** | **Management of scientific indepen-dence & conflict of interest** |
| --- | --- | --- | --- | --- | --- | --- | --- | --- | --- |
| **Austria (AUT)** | Association of Austrian Social Security Institutions (HVB)* |  |  |  |  |  |  |  |  |
| Belgium (BEL) | Belgian Statutory National Medical Insurance Association (INAMI)* |  |  |  |  |  |  |  |  |
| Bulgaria (BGR) | Ministry of Health |  |  |  |  |  |  |  |  |
| Croatia (HRV) | Croatian Institute of Health Insurance (HZZO) |  |  |  |  |  |  |  |  |
| Cyprus (CYP) | Department of Pharmaceutical Services at Ministry of Health |  |  |  |  |  |  |  |  |
| **Country** | **Institutions contacted during survey** | **Infor-mation retrieval** | **Evidence quality/**  **risk of bias** | **Publi-cation bias in analysis and synthesis** | **Identifi-cation of publi-cation bias** | **Publi-cation bias in conclusions/ recom-mendations** | **Policies for data comp-leteness** | **Manage-ment of confiden-tiality & transpa-rency** | **Management of scientific indepen-dence & conflict of interest** |
| **Czech Republic (CZR)** | Section of pricing and reimbursement regulation at State Institute for Drug Control (SÚKL) |  |  |  |  |  |  |  |  |
| Denmark (DNK) | Reimbursement Committee at the Danish Medicines Agency |  |  |  |  |  |  |  |  |
| Estonia (EST) | Ministry of Social Affairs |  |  |  |  |  |  |  |  |
| **Finland (FIN)** | Pharmaceutical Pricing Board (PPB) |  |  |  |  |  |  |  |  |
| **France (FRA)** | Transparency Commission at Haute Autorité de Santé |  |  |  |  |  |  |  |  |
| **Country** | **Institutions contacted during survey** | **Infor-mation retrieval** | **Evidence quality/**  **risk of bias** | **Publi-cation bias in analysis and synthesis** | **Identifi-cation of publi-cation bias** | **Publi-cation bias in conclusions/ recom-menda-tions** | **Policies for data comp-leteness** | **Manage-ment of confiden-tiality & transpa-rency** | **Management of scientific indepen-dence & conflict of interest** |
| **Germany (DEU)** | Federal Joint Committee (G-BA) |  |  |  |  |  |  |  |  |
| Greece (GRC) | National Organization for Medicines (EOF) |  |  |  |  |  |  |  |  |
| **Hungary (HUN)** | Reimbursement Department at National Health Insurance Fund |  |  |  |  |  |  |  |  |
| **Iceland (ISL)** | Ministry of Welfare |  |  |  |  |  |  |  |  |
| Ireland (IRL) | Health Service Executive |  |  |  |  |  |  |  |  |
| Italy (ITA) | Italian Medicines Agency (Agenzia Italiana del Farmaco) |  |  |  |  |  |  |  |  |
| **Country** | **Institutions contacted during survey** | **Infor-mation retrieval** | **Evidence quality/**  **risk of bias** | **Publi-cation bias in analysis and synthesis** | **Identifi-cation of publi-cation bias** | **Publi-cation bias in conclusions/ recom-menda-tions** | **Policies for data comp-leteness** | **Manage-ment of confiden-tiality & transpa-rency** | **Management of scientific indepen-dence & conflict of interest** |
| Latvia (LVA) | National Health Service |  |  |  |  |  |  |  |  |
| Liechtenstein (LIE) | Health Service Committee, Department of Health (Amt für Gesundheit) |  |  |  |  |  |  |  |  |
| Lithuania (LTU) | Ministry of Health* |  |  |  |  |  |  |  |  |
| Luxembourg (LUX) | Ministry of Health |  |  |  |  |  |  |  |  |
| Macedonia (MKD) | Ministry of Health* |  |  |  |  |  |  |  |  |
| **Country** | **Institutions contacted during survey** | **Infor-mation retrieval** | **Evidence quality/**  **risk of bias** | **Publi-cation bias in analysis and synthesis** | **Identifi-cation of publi-cation bias** | **Publi-cation bias in conclusions/ recom-menda-tions** | **Policies for data comp-leteness** | **Manage-ment of confiden-tiality & transpa-rency** | **Management of scientific indepen-dence & conflict of interest** |
| **Malta (MLT)** | Directorate of Pharmaceutical Policy and Monitoring at Ministry for Social Policy |  |  |  |  |  |  |  |  |
| Montenegro (MNE) | Health Insurance Fund* |  |  |  |  |  |  |  |  |
| The Netherlands (NLD) | Health Insurance Board (CVZ)* |  |  |  |  |  |  |  |  |
| Norway (NOR) | Norwegian Medicines Agency |  |  |  |  |  |  |  |  |
| Poland (POL) | Ministry of Health* |  |  |  |  |  |  |  |  |
| **Country** | **Institutions contacted during survey** | **Infor-mation retrieval** | **Evidence quality/**  **risk of bias** | **Publi-cation bias in analysis and synthesis** | **Identifi-cation of publi-cation bias** | **Publi-cation bias in conclusions/ recom-menda-tions** | **Policies for data comp-leteness** | **Manage-ment of confiden-tiality & transpa-rency** | **Management of scientific indepen-dence & conflict of interest** |
| **Portugal (PRT)** | National Authority of Medicines and Health Products (INFARMED)* |  |  |  |  |  |  |  |  |
| Romania (ROU) | Ministry of Health |  |  |  |  |  |  |  |  |
| **Serbia (SRB)** | Republic Fund of Health Insurance (RFZO) |  |  |  |  |  |  |  |  |
| Slovakia (SVK) | Ministry of Health* |  |  |  |  |  |  |  |  |
| **Slovenia (SVN)** | Health Insurance Institute of Slovenia (ZZZS) |  |  |  |  |  |  |  |  |
| **Country** | **Institutions contacted during survey** | **Infor-mation retrieval** | **Evidence quality/**  **risk of bias** | **Publi-cation bias in analysis and synthesis** | **Identifi-cation of publi-cation bias** | **Publi-cation bias in conclusions/ recom-menda-tions** | **Policies for data comp-leteness** | **Manage-ment of confiden-tiality & transpa-rency** | **Management of scientific indepen-dence & conflict of interest** |
| Spain (ESP) | Directorate of Pharmaceutical and Health Products |  |  |  |  |  |  |  |  |
| **Sweden (SWE)** | Dental and Pharmaceutical Benefits Agency (TLV) |  |  |  |  |  |  |  |  |
| Switzerland (CHE) | Federal Office of Public Health* |  |  |  |  |  |  |  |  |
| Turkey (TUR) | Social Security Institution |  |  |  |  |  |  |  |  |
| **UK - England & Wales (ENG)** | National Insitute for Health and Care Excellence |  |  |  |  |  |  |  |  |
| UK – Scotland (SCT) | National Health Service Scotland |  |  |  |  |  |  |  |  |
| Notes: Bolded country names denote survey participants; *=alternative institutions were also contacted, see additional table below.  A more compact version of the table for review purposes can be found in supplementary file 8. | | | | | | | | | |

List of institutions contacted for the survey

| **Country** | **Institution** | **Alternative Institution Contacted** |
| --- | --- | --- |
| Austria | Association of Austrian Social Security Institutions (HVB) | Gesundheit Österreich GmbH |
| Belgium | Belgian Statutory National Medical Insurance Association (INAMI) | Universität Leuven, Belgian Health Care Knowledge Centre (KCE) |
| Bulgaria | Ministry of Health |  |
| Croatia | Croatian Institute of Health Insurance |  |
| Cyprus | Department of Pharmaceutical Services at Ministry of Health |  |
| Czech Republic | Section of pricing and reimbursement regulation at State Institute for Drug Control (SÚKL) |  |
| Denmark | Reimbursement Committee at the Danish Medicines Agency |  |
| Estonia | Ministry of Social Affairs |  |
| Finland | Pharmaceutical Pricing Board (PPB) |  |
| France | Transparency Commission at Haute Autorité de Santé |  |
| Germany | Federal Joint Committee |  |
| Greece | National Organization for Medicines (EOF) |  |
| Hungary | Reimbursement Department at National Health Insurance Fund |  |
| Iceland | Ministry of Welfare |  |
| Ireland | Health Service Executive |  |
| Italy | Italian Medicines Agency (Agenzia Italiana del Farmaco) |  |
| Latvia | National Health Service |  |
| Liechtenstein | Health Service Committee, Department of Health (Amt für Gesundheit) |  |
| Lithuania | Ministry of Health | Medicines Reimbursement Department, National Health Insurance Fund |
| Luxembourg | Ministry of Health |  |
| Macedonia | Ministry of Health | University of Skopje |
| Malta | Directorate of Pharmaceutical Policy and Monitoring at Ministry for Social Policy |  |
| Montenegro | Health Insurance Fund | Ministry of Health |
| **Country** | **Institution** | **Alternative Institution Contacted** |
| The Netherlands | Health Insurance Board (CVZ) | University of Utrecht |
| Norway | Norwegian Medicines Agency |  |
| Poland | Ministry of Health | Agency for Health Technology Assessment in Poland (AOTM) |
| Portugal | National Authority of Medicines and Health Products (INFARMED) | Lisbon University |
| Romania | Ministry of Health |  |
| Serbia | Republic Fund of Health Insurance (RFZO) |  |
| Slovakia | Ministry of Health | Health Policy Institute |
| Slovenia | Health Insurance Institute of Slovenia (ZZZS) |  |
| Spain | Directorate of Pharmaceutical and Health Products |  |
| Sweden | Dental and Pharmaceutical Benefits Agency |  |
| Switzerland | Federal Office of Public Health | Swiss Insurance association |
| Turkey | Social Security Institution | Hacettepe University |
| **Country** | **Institution** | **Alternative Institution Contacted** |
| UK - England & Wales | National Insitute for Health and Care Excellence |  |
| UK - Scotland | National Health Service Scotland |  |

# 
