# Supplementary material for: Published and unpublished evidence in coverage decision-making for pharmaceuticals in Europe: existing approaches and way forward
Source: Health Res Policy Syst. 2016 Jan 26;14:6. doi: 10.1186/s12961-016-0080-9 (PMC4727332; doi:10.1186/s12961-016-0080-9)
Supplement: Additional file 2: — Search strategies for publications. (PDF 203 kb) [file 12961_2016_80_MOESM2_ESM.pdf]

Addititional file 2 Search strategies to identify publications related to institutions' policies towards publication bias (Medline, EMBASE, Cochrane Library)

Medline (Pubmed), date of search 2012-04-12

	Search	
#1	Main Association of Austrian Social Security Institutions	0
#2	Hauptverband der österreichischen Sozialversicherungsträger	3
#3	Hauptverband der osterreichischen Sozialversicherungstrager	3
#4	hauptverband der osterreichischen sozialversicherungstrager	3
#5	Hauptverband der oesterreichischen Sozialversicherungstraeger	0
#6	Heilmittel-Evaluierungs-Kommission	0
#7	Heilmittelevaluierungskommission	0
#8	Pharmaceutical Evaluation Board	213
#9	Belgian statutory national medical insurance association	0
#10	Austrian Social Security	18
#11	national medical insurance association	1130
#12	Rijksinstituut voor ziekte- en invaliditeitsverzekering	2
#13	Institut national d'assurance maladie-invalidité	1
#14	Drug Reimbursement Committee	91
#15	Commissie tegemoetkoming geneesmiddelen	0
#16	Commission de Remboursement des Médicaments	0
#17	Direction Pharmaceuticals and Medical Devices	8
#18	Department of Pharmaceutical Services	10183
#19	State Institute for Drug Control	4620
#20	Danish Medicines Agency	64
#21	Reimbursement Committee	386
#22	Lægemiddelstyrelsen	20
#23	Medicintilskudsnævnet	0
#24	Pharmaceuticals Committee	161
#25	Pharmaceutical Committee	2984
#26	Ravimiosakond	0
#27	Pharmaceuticals Pricing Board	15
#28	main association of austria social security organization	1
#29	Lääkkeiden hintalautakunta	0
#30	Läkemedelsprisnämnden	0
#31	French National Authority for Health	115
#32	Transparency Committee	128
#33	Haute Autorité de Santé	1
#34	Commission de Transparence	8
#35	Federal Joint Commitee	0
#36	federal joint committee	130
#37	Institute for Quality and Efficiency in Health Care	167
#38	Gemeinsamer Bundesausschuss	27
#39	Institut fuer Qualitaet und Wirtschaftlichkeit im Gesundheitswesen	0
#40	institut fur qualitat und wirtschaftlichkeit im gesundheitswesen	58
#41	Institut für Qualität und Wirtschaftlichkeit im Gesundheitswesen	58
#42	National Organisation for Medicines	1285
#43	product assessment division	657
#44	Human Medicines Assessment Section	23

#45	National Health Insurance Fund Administration	4638
#46	Technology Assessment Bureau	59
#47	Országos Egészségbiztosítási Pénztár	16
#48	Egészségügyi Stratégiai Kutatóintézet	0
#49	Health Service Executive	4069
#50	National Centre for Pharmacoeconomics	205
#51	Corporate Pharmaceutical Unit	5
#52	Italian Medicines Agency	25
#53	Technical and Scientific Commission	164
#54	Pricing and Reimbursement Committee	98
#55	Agenzia Italiana del Farmaco	16
#56	Commissione Tecnico Scientifica	0
#57	Comitato Prezzi e Rimborso	0
#58	Economic Evaluation of Medicinal Products Division	93
#59	Pricing and Reimbursement Committee	98
#60	Directorate of Pharmaceutical Policy and Monitoring	0
#61	Government Formulary List Advisory Committee	1
#62	Commission for pharmaceutical help	44
#63	College voor zorgverzekeringen	5
#64	Commissie Farmaceutische Hulp	1
#65	Agency for Health Technology Assessment in Poland	5
#66	National Authority of Medicines and Health Products	17
#67	Therapeutic Strategy Committee	428
#68	Agency for Medical Products and Medical Devices	108
#69	Health Insurance Institute of Slovenia	32
#70	Categorisation Commission	4
#71	Spanish Agency for Medicines and Health Products	14
#72	Spanish Agency for Medicinal Products and Medical Devices	4
#73	Spanish Agency of Medicines and Health Devices	2
#74	Spanish Agency for Medicinal Products and Health Devices	2
#75	Agencia Española de Medicamentos y Productos Sanitarios	0
#76	Inter-Ministerial Pricing Commission	0
#77	Interministerial Pricing Commission	0
#78	Dental and Pharmaceutical Benefits Agency	7
#79	Tandvårds- och läkemedelsförmånsverket	2
#80	National Institute for Clinical Excellence	1527
#81	National Institute for Health and Clinical Excellence	1264
#82	Scottish Medicines Consortium	26
#83	Reimbursement Committee	386
#84	Board of Croatian Institute for Health Insurance	3
#85	Bureau for Medicines	34
#86	Medicine Pricing and Reimbursement Committee	43
#87	Health Insurance Fund of Montenegro	1
#88	Medical and Economic Appraisal Commission	6
#89	Federal Office of Public Health	1831
#90	Federal Drug Commission	260
#91	Bundesamt für Gesundheit	35
#92	Eidgenössische Arzneimittelkommission	0
#93	Norwegian Medicines Agency	33
#94	Statens Legemiddelverk	21
#95	Medicines and Medical Devices Department	144

#96	Zāļu un medicīnisko ierīču departaments	0
#97	zalu un medicijnisko ierijcu departments	53
#98	Kompensējamo zāļu un medicīnisko ierīču nodaļa	0
#99	Zāļu ekonomiskās novērtēšanas nodaļa	0
#100	Medicines Pricing and Reimbursement Agency	16
#101	#1 OR #2 OR #3 OR #4 OR #5 OR #6 OR #7 OR #8 OR #9 OR #10 OR #11 OR #12 OR #13 OR #14 OR #15 OR #16 OR #17 OR #18 OR #19 OR #20 OR #21 OR #22 OR #23 OR #24 OR #25 OR #26 OR #27 OR #28 OR #29 OR #30 OR #31 OR #32 OR #33 OR #34 OR #35 OR #36 OR #37 OR #38 OR #39 OR #40 OR #41 OR #42 OR #43 OR #44 OR #45 OR #46 OR #47 OR #48 OR #49 OR #50 OR #51 OR #52 OR #53 OR #54 OR #55 OR #56 OR #57 OR #58 OR #59 OR #60 OR #61 OR #62 OR #63 OR #64 OR #65 OR #66 OR #67 OR #68 OR #69 OR #70 OR #71 OR #72 OR #73 OR #74 OR #75 OR #76 OR #77 OR #78 OR #79 OR #80 OR #81 OR #82 OR #83 OR #84 OR #85 OR #86 OR #87 OR #88 OR #89 OR #90 OR #91 OR #92 OR #93 OR #94 OR #95 OR #96 OR #97 OR #98 OR #99 OR #100	34877
#102	language bias[TIAB]	29
#103	citation bias[TIAB]	12
#104	location bias[TIAB]	12
#105	time lag bias[TIAB]	7
#106	retrieval bias[TIAB]	12
#107	reporting bias[TIAB]	444
#108	publication bias[TIAB]	2341
#109	"Publication Bias"[Mesh]	1760
#110	selective[TIAB]	325860
#111	unknow*[TIAB]	248605
#112	confidential*[TIAB]	10707
#113	hidden[TIAB]	16211
#114	hide[TIAB]]	1864
#115	withhold*[TIAB]	4831
#116	conceal*[TIAB]	6927
#117	disclos*[TIAB]	46224
#118	non-obtainable[TIAB]	2
#119	unobtainable[TIAB]	510
#120	nonaccessib*[TIAB]	37
#121	non-accessib*[TIAB]	41
#122	inaccessible[TIAB]	5959
#123	unavailab*[TIAB]	9921
#124	uncomplete*[TIAB]	235
#125	incomplete*[TIAB]	78062
#126	non-public*[TIAB]	131
#127	nonpublic*[TIAB]	73
#128	unpublic*[TIAB]	20
#129	unpublish*[TIAB]	7321
#130	#102 OR #103 OR #104 OR #105 OR #106 OR #107 OR #108 OR #109 OR #110 OR #111 OR #112 OR #113 OR #114 OR #115 OR #116 OR #117 OR #118 OR #119 OR #120 OR #121 OR #122 OR #123 OR #124 OR #125 OR #126 OR #127 OR #128 OR #129	749121
#131	#101 AND #130	1603
#132	Limits: Publication Date from 1994/01/01	11178897
#133	#131 AND #132	1436

EMBASE 1974 to 2012 April 25 (Ovid), date of search 2012-04-26

1 publishing/ (27317)
2 publication bias.ti,ab. (2926)
3 reporting bias.ti,ab. (547)
4 retrieval bias.ti,ab. (12)
5 time lag bias.ti,ab. (10)
6 location bias.ti,ab. (11)
7 citation bias.ti,ab. (16)
8 language bias.ti,ab. (35)
9 "unpublish*".ti,ab. (8901)
10 "unpublic*".ti,ab. (23)
11 "nonpublic*".ti,ab. (94)
12 "non-public*".ti,ab. (156)
13 "incomplete*".ti,ab. (95232)
14 "uncomplete*".ti,ab. (440)
15 "unavailab*".ti,ab. (12090)
16 inaccessible.ti,ab. (6721)
17 "non-accessib*".ti,ab. (57)
18 "nonaccessib*".ti,ab. (44)
19 unobtainable.ti,ab. (633)
20 non-obtainable.ti,ab. (2)
21 "disclos*".ti,ab. (56693)
22 "conceal*".ti,ab. (8511)
23 "withhold*".ti,ab. (5669)
24 hide.ti,ab. (2210)
25 hidden.ti,ab. (18570)
26 "confidential*".ti,ab. (12500)
27 "unknow*".ti,ab. (307679)
28 selective.ti,ab. (392039)
29 1 or 2 or 3 or 4 or 5 or 6 or 7 or 8 or 9 or 10 or 11 or 12 or 13 or 14 or 15 or 16 or 17 or 18 or 19 or 20 or 21 or 22 or 23 or 24 or 25 or 26 or 27 or 28 (934534)
30 Main Association of Austrian Social Security Institutions.mp. [mp=title, abstract, subject headings, heading word, drug trade name, original title, device manufacturer, drug manufacturer, device trade name, keyword] (1)
31 Main Association of Austrian Social Security Organisations.mp. [mp=title, abstract, subject headings, heading word, drug trade name, original title, device manufacturer, drug manufacturer, device trade name, keyword] (0)
32 Hauptverband der osterreichischen Sozialversicherungstrager.mp. [mp=title, abstract, subject headings, heading word, drug trade name, original title, device manufacturer, drug manufacturer, device trade name, keyword] (0)
33 Search Hauptverband der oesterreichischen Sozialversicherungstraeger.mp. [mp=title, abstract, subject headings, heading word, drug trade name, original title, device manufacturer, drug manufacturer, device trade name, keyword] (0)
34 Heilmittel-Evaluierungs-Kommission.mp. [mp=title, abstract, subject headings, heading word, drug trade name, original title, device manufacturer, drug manufacturer, device trade name, keyword] (0)
35 Heilmittelevaluierungskommission.mp. [mp=title, abstract, subject headings, heading word, drug trade name, original title, device manufacturer, drug manufacturer, device trade name, keyword] (0)
36 Pharmaceutical Evaluation Board.mp. [mp=title, abstract, subject headings, heading word, drug trade name, original title, device manufacturer, drug manufacturer, device trade name, keyword] (0)

37 Belgian statutory national medical insurance association.mp. [mp=title, abstract, subject headings, heading word, drug trade name, original title, device manufacturer, drug manufacturer, device trade name, keyword] (0)

38 Austrian Social Security.mp. [mp=title, abstract, subject headings, heading word, drug trade name, original title, device manufacturer, drug manufacturer, device trade name, keyword] (5)

39 national medical insurance association.mp. [mp=title, abstract, subject headings, heading word, drug trade name, original title, device manufacturer, drug manufacturer, device trade name, keyword] (0)

40 Rijksinstituut voor ziekte- en invaliditeitsverzekering.mp. [mp=title, abstract, subject headings, heading word, drug trade name, original title, device manufacturer, drug manufacturer, device trade name, keyword] (2)

41 Institut national assurance maladie-invalidite.mp. [mp=title, abstract, subject headings, heading word, drug trade name, original title, device manufacturer, drug manufacturer, device trade name, keyword] (0)

42 Drug Reimbursement Committee.mp. [mp=title, abstract, subject headings, heading word, drug trade name, original title, device manufacturer, drug manufacturer, device trade name, keyword] (4)

43 Commissie tegemoetkoming geneesmiddelen.mp. [mp=title, abstract, subject headings, heading word, drug trade name, original title, device manufacturer, drug manufacturer, device trade name, keyword] (1)

44 Commission de Remboursement des Medicaments.mp. [mp=title, abstract, subject headings, heading word, drug trade name, original title, device manufacturer, drug manufacturer, device trade name, keyword] (0)

45 Department of Pharmaceutical Services.mp. [mp=title, abstract, subject headings, heading word, drug trade name, original title, device manufacturer, drug manufacturer, device trade name, keyword] (14)

46 State Institute for Drug Control.mp. [mp=title, abstract, subject headings, heading word, drug trade name, original title, device manufacturer, drug manufacturer, device trade name, keyword] (43)

47 Danish Medicines Agency.mp. [mp=title, abstract, subject headings, heading word, drug trade name, original title, device manufacturer, drug manufacturer, device trade name, keyword] (72)

48 Reimbursement Committee.mp. [mp=title, abstract, subject headings, heading word, drug trade name, original title, device manufacturer, drug manufacturer, device trade name, keyword] (8)

49 Lagemiddelstyrelsen.mp. [mp=title, abstract, subject headings, heading word, drug trade name, original title, device manufacturer, drug manufacturer, device trade name, keyword] (0)

50 Laegemiddelstyrelsen.mp. [mp=title, abstract, subject headings, heading word, drug trade name, original title, device manufacturer, drug manufacturer, device trade name, keyword] (15)

51 Medicintilskudsaevnet.mp. [mp=title, abstract, subject headings, heading word, drug trade name, original title, device manufacturer, drug manufacturer, device trade name, keyword] (0)

52 Medicintilskudsavnet.mp. [mp=title, abstract, subject headings, heading word, drug trade name, original title, device manufacturer, drug manufacturer, device trade name, keyword] (0)

53 Pharmaceuticals Committee.mp. [mp=title, abstract, subject headings, heading word, drug trade name, original title, device manufacturer, drug manufacturer, device trade name, keyword] (1)

54 Pharmaceutical Committee.mp. [mp=title, abstract, subject headings, heading word, drug trade name, original title, device manufacturer, drug manufacturer, device trade name, keyword] (44)

55 Ravimiosakond.mp. [mp=title, abstract, subject headings, heading word, drug trade name, original title, device manufacturer, drug manufacturer, device trade name, keyword] (0)

56 Pharmaceuticals Pricing Board.mp. [mp=title, abstract, subject headings, heading word, drug trade name, original title, device manufacturer, drug manufacturer, device trade name, keyword] (0)

57 Pharmaceutical Pricing Board.mp. [mp=title, abstract, subject headings, heading word, drug trade name, original title, device manufacturer, drug manufacturer, device trade name, keyword] (0)

58 main association of austria social security organization.mp. [mp=title, abstract, subject headings, heading word, drug trade name, original title, device manufacturer, drug manufacturer, device trade name, keyword] (0)

59 Laakkeiden hintalautakunta.mp. [mp=title, abstract, subject headings, heading word, drug trade name, original title, device manufacturer, drug manufacturer, device trade name, keyword] (0)

60 hintalautakunta.mp. [mp=title, abstract, subject headings, heading word, drug trade name, original title, device manufacturer, drug manufacturer, device trade name, keyword] (0)

61 Laeaekkeiden.mp. [mp=title, abstract, subject headings, heading word, drug trade name, original title, device manufacturer, drug manufacturer, device trade name, keyword] (1)

62 Lakemedelsprisnamnden.mp. [mp=title, abstract, subject headings, heading word, drug trade name, original title, device manufacturer, drug manufacturer, device trade name, keyword] (0)

63 Laekemedelsprisnaemnden.mp. [mp=title, abstract, subject headings, heading word, drug trade name, original title, device manufacturer, drug manufacturer, device trade name, keyword] (0)

64 French National Authority for Health.mp. [mp=title, abstract, subject headings, heading word, drug trade name, original title, device manufacturer, drug manufacturer, device trade name, keyword] (67)

65 Transparency Committee.mp. [mp=title, abstract, subject headings, heading word, drug trade name, original title, device manufacturer, drug manufacturer, device trade name, keyword] (20)

66 Haute Autorite de Sante.mp. [mp=title, abstract, subject headings, heading word, drug trade name, original title, device manufacturer, drug manufacturer, device trade name, keyword] (138)

67 Commission de Transparence.mp. [mp=title, abstract, subject headings, heading word, drug trade name, original title, device manufacturer, drug manufacturer, device trade name, keyword] (7)

68 Federal Joint Commitee.mp. [mp=title, abstract, subject headings, heading word, drug trade name, original title, device manufacturer, drug manufacturer, device trade name, keyword] (1)

69 federal joint committee.mp. [mp=title, abstract, subject headings, heading word, drug trade name, original title, device manufacturer, drug manufacturer, device trade name, keyword] (91)

70 (Institute for Quality and Efficiency in Health Care).mp. [mp=title, abstract, subject headings, heading word, drug trade name, original title, device manufacturer, drug manufacturer, device trade name, keyword] (96)

71 (Direction Pharmaceuticals and Medical Devices).mp. [mp=title, abstract, subject headings, heading word, drug trade name, original title, device manufacturer, drug manufacturer, device trade name, keyword] (0)

72 Gemeinsamer Bundesausschuss.mp. [mp=title, abstract, subject headings, heading word, drug trade name, original title, device manufacturer, drug manufacturer, device trade name, keyword] (33)

73 Institut fuer Qualitaet und Wirtschaftlichkeit im Gesundheitswesen.mp. [mp=title, abstract, subject headings, heading word, drug trade name, original title, device manufacturer, drug manufacturer, device trade name, keyword] (0)

74 institut fur qualitat und wirtschaftlichkeit im gesundheitswesen.mp. [mp=title, abstract, subject headings, heading word, drug trade name, original title, device manufacturer, drug manufacturer, device trade name, keyword] (27)

75 National Organisation for Medicines.mp. [mp=title, abstract, subject headings, heading word, drug trade name, original title, device manufacturer, drug manufacturer, device trade name, keyword] (2)

76 product assessment division.mp. [mp=title, abstract, subject headings, heading word, drug trade name, original title, device manufacturer, drug manufacturer, device trade name, keyword] (0)

77 Human Medicines Assessment Section.mp. [mp=title, abstract, subject headings, heading word, drug trade name, original title, device manufacturer, drug manufacturer, device trade name, keyword] (0)

78 National Health Insurance Fund Administration.mp. [mp=title, abstract, subject headings, heading word, drug trade name, original title, device manufacturer, drug manufacturer, device trade name, keyword] (73)

79 Technology Assessment Bureau.mp. [mp=title, abstract, subject headings, heading word, drug trade name, original title, device manufacturer, drug manufacturer, device trade name, keyword] (0)

80 Orszagos Egeszsegbiztositasi Penztar.mp. [mp=title, abstract, subject headings, heading word, drug trade name, original title, device manufacturer, drug manufacturer, device trade name, keyword] (0)

81 Egeszsegugyi Strategiai Kutatointezet.mp. [mp=title, abstract, subject headings, heading word, drug trade name, original title, device manufacturer, drug manufacturer, device trade name, keyword] (0)

82 Health Service Executive.mp. [mp=title, abstract, subject headings, heading word, drug trade name, original title, device manufacturer, drug manufacturer, device trade name, keyword] (108)

83 National Centre for Pharmacoeconomics.mp. [mp=title, abstract, subject headings, heading word, drug trade name, original title, device manufacturer, drug manufacturer, device trade name, keyword] (6)

84 Corporate Pharmaceutical Unit.mp. [mp=title, abstract, subject headings, heading word, drug trade name, original title, device manufacturer, drug manufacturer, device trade name, keyword] (0)

85 Italian Medicines Agency.mp. [mp=title, abstract, subject headings, heading word, drug trade name, original title, device manufacturer, drug manufacturer, device trade name, keyword] (20)

86 (Technical and Scientific Commission).mp. [mp=title, abstract, subject headings, heading word, drug trade name, original title, device manufacturer, drug manufacturer, device trade name, keyword] (1)

87 (Pricing and Reimbursement Committee).mp. [mp=title, abstract, subject headings, heading word, drug trade name, original title, device manufacturer, drug manufacturer, device trade name, keyword] (1)

88 Agenzia Italiana del Farmaco.mp. [mp=title, abstract, subject headings, heading word, drug trade name, original title, device manufacturer, drug manufacturer, device trade name, keyword] (20)

89 Commissione Tecnico Scientifica.mp. [mp=title, abstract, subject headings, heading word, drug trade name, original title, device manufacturer, drug manufacturer, device trade name, keyword] (1)

90 Comitato Prezzi e Rimborso.mp. [mp=title, abstract, subject headings, heading word, drug trade name, original title, device manufacturer, drug manufacturer, device trade name, keyword] (0)

91 Economic Evaluation of Medicinal Products Division.mp. [mp=title, abstract, subject headings, heading word, drug trade name, original title, device manufacturer, drug manufacturer, device trade name, keyword] (0)

92 (Pricing and Reimbursement Committee).mp. [mp=title, abstract, subject headings, heading word, drug trade name, original title, device manufacturer, drug manufacturer, device trade name, keyword] (1)

93 (Directorate of Pharmaceutical Policy and Monitoring).mp. [mp=title, abstract, subject headings, heading word, drug trade name, original title, device manufacturer, drug manufacturer, device trade name, keyword] (0)

94 Government Formulary List Advisory Committee.mp. [mp=title, abstract, subject headings, heading word, drug trade name, original title, device manufacturer, drug manufacturer, device trade name, keyword] (0)

95 Commission for pharmaceutical help.mp. [mp=title, abstract, subject headings, heading word, drug trade name, original title, device manufacturer, drug manufacturer, device trade name, keyword] (2)

96 College voor zorgverzekeringen.mp. [mp=title, abstract, subject headings, heading word, drug trade name, original title, device manufacturer, drug manufacturer, device trade name, keyword] (4)

97 Commissie Farmaceutische Hulp.mp. [mp=title, abstract, subject headings, heading word, drug trade name, original title, device manufacturer, drug manufacturer, device trade name, keyword] (0)

98 Agency for Health Technology Assessment in Poland.mp. [mp=title, abstract, subject headings, heading word, drug trade name, original title, device manufacturer, drug manufacturer, device trade name, keyword] (8)

99 (National Authority of Medicines and Health Products).mp. [mp=title, abstract, subject headings, heading word, drug trade name, original title, device manufacturer, drug manufacturer, device trade name, keyword] (0)

100 Therapeutic Strategy Committee.mp. [mp=title, abstract, subject headings, heading word, drug trade name, original title, device manufacturer, drug manufacturer, device trade name, keyword] (0)

101 Agencja Oceny Technologii Medycznych.mp. [mp=title, abstract, subject headings, heading word, drug trade name, original title, device manufacturer, drug manufacturer, device trade name, keyword] (0)

102 (Agency for Medical Products and Medical Devices).mp. [mp=title, abstract, subject headings, heading word, drug trade name, original title, device manufacturer, drug manufacturer, device trade name, keyword] (0)

103 Health Insurance Institute of Slovenia.mp. [mp=title, abstract, subject headings, heading word, drug trade name, original title, device manufacturer, drug manufacturer, device trade name, keyword] (8)

104 Categorisation Commission.mp. [mp=title, abstract, subject headings, heading word, drug trade name, original title, device manufacturer, drug manufacturer, device trade name, keyword] (0)

105 Categorization Commission.mp. [mp=title, abstract, subject headings, heading word, drug trade name, original title, device manufacturer, drug manufacturer, device trade name, keyword] (0)

106 (Spanish Agency for Medicines and Health Products).mp. [mp=title, abstract, subject headings, heading word, drug trade name, original title, device manufacturer, drug manufacturer, device trade name, keyword] (3)

107 (Spanish Agency for Medicinal Products and Medical Devices).mp. [mp=title, abstract, subject headings, heading word, drug trade name, original title, device manufacturer, drug manufacturer, device trade name, keyword] (1)

108 (Spanish Agency of Medicines and Health Devices).mp. [mp=title, abstract, subject headings, heading word, drug trade name, original title, device manufacturer, drug manufacturer, device trade name, keyword] (0)

109 (Spanish Agency for Medicinal Products and Health Devices).mp. [mp=title, abstract, subject headings, heading word, drug trade name, original title, device manufacturer, drug manufacturer, device trade name, keyword] (0)

110 Agencia Espanola de Medicamentos y Productos Sanitarios.mp. [mp=title, abstract, subject headings, heading word, drug trade name, original title, device manufacturer, drug manufacturer, device trade name, keyword] (3)

111 Inter-Ministerial Pricing Commission.mp. [mp=title, abstract, subject headings, heading word, drug trade name, original title, device manufacturer, drug manufacturer, device trade name, keyword] (0)

112 Interministerial Pricing Commission.mp. [mp=title, abstract, subject headings, heading word, drug trade name, original title, device manufacturer, drug manufacturer, device trade name, keyword] (0)

113 (Dental and Pharmaceutical Benefits Agency).mp. [mp=title, abstract, subject headings, heading word, drug trade name, original title, device manufacturer, drug manufacturer, device trade name, keyword] (6)

114 Tandvards- och lakemedelsformansverket.mp. [mp=title, abstract, subject headings, heading word, drug trade name, original title, device manufacturer, drug manufacturer, device trade name, keyword] (3)

115 laekemedelsformansverket.mp. [mp=title, abstract, subject headings, heading word, drug trade name, original title, device manufacturer, drug manufacturer, device trade name, keyword] (0)
116 National Institute for Clinical Excellence.mp. [mp=title, abstract, subject headings, heading word, drug trade name, original title, device manufacturer, drug manufacturer, device trade name, keyword] (835)
117 (National Institute for Health and Clinical Excellence).mp. [mp=title, abstract, subject headings, heading word, drug trade name, original title, device manufacturer, drug manufacturer, device trade name, keyword] (1284)
118 Scottish Medicines Consortium.mp. [mp=title, abstract, subject headings, heading word, drug trade name, original title, device manufacturer, drug manufacturer, device trade name, keyword] (58)
119 Reimbursement Committee.mp. [mp=title, abstract, subject headings, heading word, drug trade name, original title, device manufacturer, drug manufacturer, device trade name, keyword] (8)
120 Board of Croatian Institute for Health Insurance.mp. [mp=title, abstract, subject headings, heading word, drug trade name, original title, device manufacturer, drug manufacturer, device trade name, keyword] (0)
121 Bureau for Medicines.mp. [mp=title, abstract, subject headings, heading word, drug trade name, original title, device manufacturer, drug manufacturer, device trade name, keyword] (2)
122 (Medicine Pricing and Reimbursement Committee).mp. [mp=title, abstract, subject headings, heading word, drug trade name, original title, device manufacturer, drug manufacturer, device trade name, keyword] (0)
123 Health Insurance Fund of Montenegro.mp. [mp=title, abstract, subject headings, heading word, drug trade name, original title, device manufacturer, drug manufacturer, device trade name, keyword] (2)
124 (Medicines Pricing and Reimbursement Committee).mp. [mp=title, abstract, subject headings, heading word, drug trade name, original title, device manufacturer, drug manufacturer, device trade name, keyword] (0)
125 (Medical and Economic Appraisal Commission).mp. [mp=title, abstract, subject headings, heading word, drug trade name, original title, device manufacturer, drug manufacturer, device trade name, keyword] (0)
126 Federal Office of Public Health.mp. [mp=title, abstract, subject headings, heading word, drug trade name, original title, device manufacturer, drug manufacturer, device trade name, keyword] (99)
127 Federal Drug Commission.mp. [mp=title, abstract, subject headings, heading word, drug trade name, original title, device manufacturer, drug manufacturer, device trade name, keyword] (0)
128 Bundesamt fur Gesundheit.mp. [mp=title, abstract, subject headings, heading word, drug trade name, original title, device manufacturer, drug manufacturer, device trade name, keyword] (0)
129 Bundesamt fuer Gesundheit.mp. [mp=title, abstract, subject headings, heading word, drug trade name, original title, device manufacturer, drug manufacturer, device trade name, keyword] (0)
130 Eidgenossische Arzneimittelkommission.mp. [mp=title, abstract, subject headings, heading word, drug trade name, original title, device manufacturer, drug manufacturer, device trade name, keyword] (0)
131 Eidgenoessische Arzneimittelkommission.mp. [mp=title, abstract, subject headings, heading word, drug trade name, original title, device manufacturer, drug manufacturer, device trade name, keyword] (0)
132 Norwegian Medicines Agency.mp. [mp=title, abstract, subject headings, heading word, drug trade name, original title, device manufacturer, drug manufacturer, device trade name, keyword] (24)
133 Statens Legemiddelverk.mp. [mp=title, abstract, subject headings, heading word, drug trade name, original title, device manufacturer, drug manufacturer, device trade name, keyword] (6)
134 (Medicines and Medical Devices Department).mp. [mp=title, abstract, subject headings, heading word, drug trade name, original title, device manufacturer, drug manufacturer, device trade name, keyword] (0)
135 Zalu un medicinisko iericu departaments.mp. [mp=title, abstract, subject headings, heading word, drug trade name, original title, device manufacturer, drug manufacturer, device trade name, keyword] (0)
136 zalu un medicijnisko ierijcu departments.mp. [mp=title, abstract, subject headings, heading word, drug trade name, original title, device manufacturer, drug manufacturer, device trade name, keyword] (0)
137 Kompensejamo zalu un medicinisko iercu nodala.mp. [mp=title, abstract, subject headings, heading word, drug trade name, original title, device manufacturer, drug manufacturer, device trade name, keyword] (0)
138 Kompensejamo zalu un medicijnisko ierijcu nodala.mp. [mp=title, abstract, subject headings, heading word, drug trade name, original title, device manufacturer, drug manufacturer, device trade name, keyword] (0)
139 Kompensejamo zalu un medicinisko iericu nodala.mp. [mp=title, abstract, subject headings, heading word, drug trade name, original title, device manufacturer, drug manufacturer, device trade name, keyword] (0)
140 Zalu ekonomiskas novertesanas nodala.mp. [mp=title, abstract, subject headings, heading word, drug trade name, original title, device manufacturer, drug manufacturer, device trade name, keyword] (0)

141 (Medicines Pricing and Reimbursement Agency).mp. [mp=title, abstract, subject headings, heading word, drug trade name, original title, device manufacturer, drug manufacturer, device trade name, keyword] (0)

142 30 or 31 or 32 or 33 or 34 or 35 or 36 or 37 or 38 or 39 or 40 or 41 or 42 or 43 or 44 or 45 or 46 or 47 or 48 or 49 or 50 or 51 or 52 or 53 or 54 or 55 or 56 or 57 or 58 or 59 or 60 or 61 or 62 or 63 or 64 or 65 or 66 or 67 or 68 or 69 or 70 or 71 or 72 or 73 or 74 or 75 or 76 or 77 or 78 or 79 or 80 or 81 or 82 or 83 or 84 or 85 or 86 or 87 or 88 or 89 or 90 or 91 or 92 or 93 or 94 or 95 or 96 or 97 or 98 or 99 or 100 or 101 or 102 or 103 or 104 or 105 or 106 or 107 or 108 or 109 or 110 or 111 or 112 or 113 or 114 or 115 or 116 or 117 or 118 or 119 or 120 or 121 or 122 or 123 or 124 or 125 or 126 or 127 or 128 or 129 or 130 or 131 or 132 or 133 or 134 or 135 or 136 or 137 or 138 or 139 or 140 or 141 (3128)

143 29 and 142 (204)

144 limit 143 to yr="1994 -Current" (202)

145 143 and 144 (202)

Cochrane Library **(all databases), date of search 2012-04-26**

#1 MeSH descriptor Publication Bias explode all trees 136

#2 MeSH descriptor Duplicate Publication as Topic explode all trees 1

#3 citation bias:ti,ab 936

#4 language bias:ti,ab 3508

#5 location bias:ti,ab 540

#6 publication bias:ti,ab 6111

#7 reporting bias:ti,ab 2700

#8 time lag bias:ti,ab 74

#9 retrieval bias:ti,ab 325

#10 selective:ti,ab 10078

#11 unknow*:ti,ab 6557

#12 confidential*:ti,ab 227

#13 hidden:ti,ab 241

#14 hide:ti,ab 241

#15 withhold*:ti,ab 209

#16 conceal*:ti,ab 1073

#17 disclos*:ti,ab 1478

#18 non-obtainable:ti,ab 0

#19 unobtainable:ti,ab 11

#20 nonaccessib*:ti,ab 1

#21 non-accessib*:ti,ab 2

#22 inaccessible:ti,ab 62

#23 unavailab*:ti,ab 336

#24 uncomplete*:ti,ab 11

#25 incomplete*:ti,ab 2489

#26 non-public*:ti,ab 21

#27 nonpublic*:ti,ab 14

#28 unpublic*:ti,ab 0

#29 unpublish*:ti,ab 1066

#30 (#1 OR #2 OR #3 OR #4 OR #5 OR #6 OR #7 OR #8 OR #9 OR #10 OR #11 OR #12 OR #13 OR #14 OR #15 OR #16 OR #17 OR #18 OR #19 OR #20 OR #21 OR #22 OR #23 OR #24 OR #25 OR #26 OR #27 OR #28 OR #29) 28304

#31 Main Association of Austrian Social Security Institutions 1

#32 Hauptverband der österreichischen Sozialversicherungsträger 0

#33 Hauptverband der osterreichischen Sozialversicherungstrager 0

#34 Hauptverband der oesterreichischen Sozialversicherungstraeger 0

#35	Heilmittel-Evaluierungs-Kommission	0
#36	Heilmittelevaluierungskommission	0
#37	Pharmaceutical Evaluation Board	365
#38	Belgian statutory national medical insurance association	0
#39	Austrian Social Security	4
#40	National Medical Insurance Association	279
#41	Rijksinstituut voor ziekte- en invaliditeitsverzekering	1
#42	Institut national d'assurance maladie-invalidité	0
#43	Drug Reimbursement Committee	73
#44	Commissie tegemoetkoming geneesmiddelen	0
#45	Commission de Remboursement des Médicaments	0
#46	Direction Pharmaceuticals and Medical Devices	188
#47	Department of Pharmaceutical Services	934
#48	State Institute for Drug Control	5839
#49	Danish Medicines Agency	116
#50	Reimbursement Committee	99
#51	Lægemiddelstyrelsen	0
#52	Commission de Remboursement des Medicaments	0
#53	Laegemiddelstyrelsen	0
#54	Medicintilskudsnævnet	0
#55	Medicintilskudsnaevnet	0
#56	Pharmaceuticals Committee	830
#57	Pharmaceutical Committee	830
#58	Ravimiosakond	0
#59	Pharmaceuticals Pricing Board	90
#60	Main Association of Austria Social Security Organizations	12
#61	Main Association of Austria Social Security Organisations	12
#62	Lääkkeiden hintalautakunta	0
#63	Laakkeiden hintalautakunta	0
#64	hintalautakunta	0
#65	Läkemedelsprisnämnden	0
#66	Lakemedelsprisnamnden	0
#67	French National Authority for Health	204
#68	Transparency Committee	49
#69	Haute Autorité de Santé	3
#70	Haute Autorite de Sante	91
#71	Commission de Transparence	1
#72	Federal Joint Commitee	2
#73	Federal Joint Committee	91
#74	Institute for Quality and Efficiency in Health Care	549
#75	Gemeinsamer Bundesausschuss	2
#76	Institut fuer Qualitaet und Wirtschaftlichkeit im Gesundheitswesen	78
#77	Institut fur Qualitat und Wirtschaftlichkeit im Gesundheitswesen	3
#78	Institut für Qualität und Wirtschaftlichkeit im Gesundheitswesen	4
#79	National Organisation for Medicines	2809
#80	Product Assessment Division	339
#81	Human Medicines Assessment Section	4302
#82	National Health Insurance Fund Administration	228
#83	Technology Assessment Bureau	354
#84	Országos Egészségbiztosítási Pénztár	0
#85	Orszagos Egeszsegbiztositasi Penztar	0
#86	Egészségügyi Stratégiai Kutatóintézet	0

#87	Egeszsegugyi Strategiai Kutatointezet	0
#88	Egeszseguegyi Strategiai Kutatointezet	0
#89	Health Service Executive	538
#90	National Centre for Pharmacoeconomics	459
#91	Corporate Pharmaceutical Unit	12
#92	Italian Medicines Agency	143
#93	Technical and Scientific Commission	60
#94	Pricing and Reimbursement Committee	40
#95	Agenzia Italiana del Farmaco	12
#96	Commissione Tecnico Scientifica	0
#97	Comitato Prezzi e Rimborso	0
#98	Economic Evaluation of Medicinal Products Division	9
#99	Pricing and Reimbursement Committee	40
#100	Directorate of Pharmaceutical Policy and Monitoring	18
#101	Government Formulary List Advisory Committee	1
#102	Commission for pharmaceutical help	112
#103	College voor zorgverzekeringen	146
#104	Commissie Farmaceutische Hulp	0
#105	Agency for Health Technology Assessment in Poland	53
#106	Agencja Oceny Technologii Medycznych	0
#107	National Authority of Medicines and Health Products	186
#108	Therapeutic Strategy Committee	1317
#109	Agency for Medical Products and Medical Devices	156
#110	Health Insurance Institute of Slovenia	10
#111	Categorisation Commission	29
#112	Categorization Commission	29
#113	Spanish Agency for Medicines and Health Products	80
#114	Spanish Agency for Medicinal Products and Medical Devices	3
#115	Spanish Agency of Medicines and Health Devices	68
#116	Spanish Agency for Medicinal Products and Health Devices	3
#117	Agencia Española de Medicamentos y Productos Sanitarios	0
#118	Agencia Espanola de Medicamentos y Productos Sanitarios	0
#119	Inter-Ministerial Pricing Commission	0
#120	Interministerial Pricing Commission	0
#121	Dental and Pharmaceutical Benefits Agency	40
#122	Tandvårds- och Läkemedelsförmånsverket	0
#123	Tandvards- och Lakemedelsformansverket	0
#124	Tandvards- och Laekemedelsfoermansverket	0
#125	National Institute for Clinical Excellence	1396
#126	National Institute for Health and Clinical Excellence	1370
#127	Scottish Medicines Consortium	27
#128	Reimbursement Committee	99
#129	Board of Croatian Institute for Health Insurance	2
#130	Bureau for Medicines	257
#131	Medicine Pricing and Reimbursement Committee	28
#132	Health Insurance Fund of Montenegro	6
#133	Medical and Economic Appraisal Commission	62
#134	Federal Office of Public Health	202
#135	Federal Drug Commission	69
#136	Bundesamt für Gesundheit	1
#137	Bundesamt fur Gesundheit	0
#138	Eidgenössische Arzneimittelkommission	0

#139 Eidgenossische Arzneimittelkommission 0

#140 Eidgenoessische Arzneimittelkommission 0

#141 Norwegian Medicines Agency 93

#142 Statens Legemiddelverk 1

#143 Medicines and Medical Devices Department 2392

#144 zalu un medicijnisko ierijcu departments 0

#145 Kompensejamo zalu un medicijnisko ierijcu nodala 0

#146 Zalu ekonomiskas novertesanas nodala 0

#147 Medicines Pricing and Reimbursement Agency 43

#148 (#31 OR #32 OR #33 OR #34 OR #35 OR #36 OR #37 OR #38 OR #39 OR #40 OR #41 OR #42 OR #43 OR #44 OR #45 OR #46 OR #47 OR #48 OR #49 OR #50 OR #51 OR #52 OR #53 OR #54 OR #55 OR #56 OR #57 OR #58 OR #59 OR #60 OR #61 OR #62 OR #63 OR #64 OR #65 OR #66 OR #67 OR #68 OR #69 OR #70 OR #71 OR #72 OR #73 OR #74 OR #75 OR #76 OR #77 OR #78 OR #79 OR #80 OR #81 OR #82 OR #83 OR #84 OR #85 OR #86 OR #87 OR #88 OR #89 OR #90 OR #91 OR #92 OR #93 OR #94 OR #95 OR #96 OR #97 OR #98 OR #99 OR #100 OR #101 OR #102 OR #103 OR #104 OR #105 OR #106 OR #107 OR #108 OR #109 OR #110 OR #111 OR #112 OR #113 OR #114 OR #115 OR #116 OR #117 OR #118 OR #119 OR #120 OR #121 OR #122 OR #123 OR #124 OR #125 OR #126 OR #127 OR #128 OR #129 OR #130 OR #131 OR #132 OR #133 OR #134 OR #135 OR #136 OR #137 OR #138 OR #139 OR #140 OR #141 OR #142 OR #143 OR #144 OR #145 OR #146 OR #147) 13555

#149 (#30 AND #148) 2288

#150 <nothing>, from 1994 to 2012 522662

#151 (#149 AND #150) 2266

There are **1997** results out of **7227 records**
