# Supplementary material for: Published and unpublished evidence in coverage decision-making for pharmaceuticals in Europe: existing approaches and way forward
Source: Health Res Policy Syst. 2016 Jan 26;14:6. doi: 10.1186/s12961-016-0080-9 (PMC4727332; doi:10.1186/s12961-016-0080-9)
Supplement: Additional file 3: Table S2. — Inclusion criteria for regulatory documents and publications. (DOCX 18 kb) [file 12961_2016_80_MOESM3_ESM.docx]

# Table 2 Inclusion criteria for regulatory documents and publications

| **Inclusion criteria for regulatory documents** | |
| --- | --- |
| **1** | **Regulatory document^a^ referring to regulatory institution included in the analysis** |
| **2** | **Document provides detailed description of methodology or process for benefit assessment of pharmaceuticals for public reimbursement or pricing** |
| **3** | **Document provides information on pre-defined aspects related to unpublished or incomplete data (see Box 1)** |
| **4** | **No newer version of the document available** |
| **5** | **Document in English, Bulgarian, Croatian, Czech, Danish, Dutch, French, German, Greek, Italian, Macedonian, Norwegian, Polish, Romanian, Russian, Serbian, Slovakian, Slovenian, Spanish or Swedish** |
| **6** | **Full text version available^b^** |
| **Inclusion criteria for publications** | |
| **1** | **Publication refers to methodology or process of pharmaceutical benefit assessment by a regulatory institution included in the analysis** |
| **2** | **Publication provides information on pre-defined aspects related to unpublished or incomplete data (see Box 1)** |
| **3** | **Publication in English, Bulgarian, Croatian, Czech, Danish, Dutch, French, German, Greek, Italian, Macedonian, Norwegian, Polish, Romanian, Russian, Serbian, Slovakian, Slovenian, Spanish or Swedish** |
| **4** | **Full text version available**^b^ |
| **5** | **published after 1993^c^** |
| a e.g. guideline, directive, code of procedure, law, provision, decree, official statement, statute, declaration, regulation, recommendation, guidance, manual, submission requirements, submission templates, description of methods, etc. (published or unpublished)  b available, complete and final version of document  c year of establishment of institutions /bodies determining the value of pharmaceuticals for public reimbursement and/or pricing purposes in European countries | |
