# Supplementary material for: Published and unpublished evidence in coverage decision-making for pharmaceuticals in Europe: existing approaches and way forward
Source: Health Res Policy Syst. 2016 Jan 26;14:6. doi: 10.1186/s12961-016-0080-9 (PMC4727332; doi:10.1186/s12961-016-0080-9)
Supplement: Additional file 4: — Survey tool used among participating institutions. (PDF 369 kb) [file 12961_2016_80_MOESM4_ESM.pdf]

Additional File 4 Survey tool used among participating institutions

# Survey tool

## Introduction

The objective of this survey is to compile policies towards publication bias that are applied by European regulatory institutions in charge of benefit assessments of pharmaceuticals in the context of reimbursement and pricing decisions. Benefit assessments, also named clinical effectiveness assessments, postlicensing evaluations or health technology assessments (HTA), are increasingly used as an essential factor in determining reimbursement and pricing of pharmaceuticals in European countries and elsewhere. Generally speaking, funding agencies and policymakers seek to make healthcare decisions based on the entire relevant research evidence base. However, this can frequently not be achieved, to a large part because a considerable amount of research findings are or not published or are incomplete.

The following survey consists of two parts:

In Part A we kindly ask you to fill in a questionnaire addressing your institution's policy towards unpublished or incomplete data.

In Part B we kindly ask you to review and supplement drafted information to describe the process of benefit assessment and decisionmaking on reimbursement and pricing of pharmaceuticals in your country and to provide supplementary material addressing institutions' policy towards unpublished or incomplete data in your country.

Required time approx. 45-60 min.

We thank you very much for your participation!

## PART A: Institutional policy towards unpublished or incomplete data.

In Part A of the survey we kindly ask you to fill in a questionnaire addressing your institution's policy towards unpublished or incomplete data. In order to facilitate filling in the questionnaire, most answers are prephrased. At few points of the questionnaire, however, we kindly ask you to provide some written comments. Please click NEXT to start the questionnaire.

### 1. What kind of scientific evidence is considered for benefit assessments in the context of reimbursement or pricing of pharmaceuticals at your institution?

| | Yes | No | I do not know |
|---|---|---|---|
| Published evidence | | | |
| Unpublished evidence | | | |

Comments (free text box):

2. **In order to avoid bias related to unpublished or incomplete data: Which of the following methodological approaches does your institution apply or require in benefit assessments of pharmaceuticals? (Please tick all options that apply)**

- Making efforts to retrieve complete data
- Assessing risk of bias due to unpublished or incomplete data
- Considering unpublished or incomplete data in data synthesis
- Considering unpublished or incomplete data in interpretation, conclusion or recommendation

3. **The retrieval of information for benefit assessments of pharmaceuticals carried out or required by your institution …**

| | Yes | No | I do not know |
|---|---|---|---|
| … attempts to identify all relevant scientific evidence. | | | |

Comments (free text box):

4. **The retrieval of information for benefit assessments of pharmaceuticals carried out or required by your institution …**

| | Yes | No | I do not know |
|---|---|---|---|
| … requires a systematic search for scientific evidence (i.e. according to principles of evidence-based medicine). | | | |
| … uses a nonsystematic approach to retrieve scientific evidence (e.g. submission of a limited number of pivotal studies). | | | |
| ... does not include scientific evidence. | | | |

Comments (free text box):

5. **Is the retrieval of information for benefit assessments of pharmaceuticals regularly restricted to publications in specific languages? (choose applicable answer)**

- No
- Yes, to English
- Yes, to non-English language(s) of your country
- Yes, to English and to language(s) of your country
- Other (please specify)

## 6. What sources are used to retrieve information for benefit assessments of pharmaceuticals? Documents/data derived from …

| | Always | Occasionally | Never |
|---|---|---|---|
| …published scientific literature | | | |
| …manufacturers or sponsors | | | |
| …national regulatory agencies for marketing authorisation | | | |
| …European Medicines Agency (EMA) | | | |
| …Food and Drug Administration (FDA) | | | |
| …clinical trial (meta-)registries | | | |
| …disease registries | | | |
| … study authors, researchers, or investigators | | | |
| …other benefit assessment/HTA institutions in your own country (at national, regional or local level) | | | |
| …other public bodies in your own country (e.g. ministry, committees) | | | |
| …benefit assessment/HTA institutions in other European countries | | | |
| …benefit assessment institutions outside Europe | | | |
| …European or international networks of assessment bodies/HTA institutions (e.g. HEN, INAHTA, EUnetHTA, etc.) | | | |
| …clinical guidelines | | | |
| …patients/consumers or their associations | | | |
| …healthcare professionals or their associations (e.g. physicians, pharmacists) | | | |
| … hospitals or their associations | | | |
| …payers or their associations | | | |

Comments (free text box):

IF published scientific literature is used as source of information for benefit assessments of pharmaceuticals, please answer the following questions. IF NOT, please continue with the NEXT page.

## 7. Which bibliographic databases are required to be searched in order to identify information on pharmaceutical benefit? (Please tick all options that apply)

- Medline
- Embase
- Cochrane Library
- CINAHL
- PsychINFO
- TRIP-Database
- CRD Databases (DARE, NHS-EED, HTA-Database)
- Other (please specify)

### 8. Bibliographic database search is required to be supplemented by … (Please tick all options that apply)

- … hand search of reference lists
- … hand search in selected relevant journals
- … search for grey literature (e.g. academic papers, research reports, governmental reports, business and industry document, etc.)
- ... search for part published studies (e.g. congress or conference abstracts, posters, proceedings)
- There is no requirement to supplement bibliographic database search.
- Other (please specify)

IF information from clinical trial (meta) registries is used as source for benefit assessments of pharmaceuticals, please answer the following questions. IF NOT, please continue with the NEXT page.

### 9. Which clinical trial registries or meta-registries are required to be searched in order to identify information on pharmaceutical benefit? (Please tick all options that apply)

- EU Clinical Trials Register (www.clinicaltrialsregister.eu)
- Clinical Trial Register provided by U.S. National Institutes of Health (www.clinicaltrials.gov)
- WHO International Clinical Trials Registry Platform (www.who.int/ictrp)
- Register provided by Current Controlled Trials Ltd.(www.controlled-trials.com)
- Registries provided by pharmaceutical companies
- Other (please specify) – CENTRAL

IF information from marketing authorisation agencies is used as source for benefit assessments of pharmaceuticals, please answer the following question. IF NOT, please continue with the NEXT page.

### 10. What kind of information from marketing authorisation agencies is required for benefit assessments of pharmaceuticals? (Please tick all options that apply)

- Study protocols of marketing authorisation studies
- Clinical study reports of marketing authorisation studies
- European or national public assessment report (EPAR/NEPAR)
- Opinion of Committee for Medicinal Products for Human Use (CHMP)
- Periodic Safety Update Reports (PSUR)
- Information from EudraVigilance
- Food and Drug Administration (FDA) Medical Review
- Information from FDA Adverse Event Reporting System (AERS)
- Other (please specify)

IF information from manufacturers or sponsors is used as source for benefit assessments of pharmaceuticals, please answer the following question. IF NOT continue with the NEXT page.

### 11. What information are manufactures or sponsors required to provide for benefit assessments of pharmaceuticals? Relevant information on … (Please tick all options that apply)

- … all published clinical studies
- … all unpublished clinical studies
- … all completed clinical studies
- … all ongoing clinical studies
- … all updated analysis of clinical studies
- … all clinical studies sponsored by them
- … all clinical studies sponsored by others
- Other (please specify)

### 12. Is retrieved evidence on pharmaceutical benefit checked for risk of publication bias (i.e. bias related to publication of studies based on the direction or strength of results)?

- Yes
- No
- I do not know

If YES, please outline briefly how this is done or give reference. If possible, please additionally provide practical examples or give reference to practical examples.

### 13. Is retrieved evidence checked for risk of selective reporting / outcome reporting bias (i.e. bias related to part-published studies)?

- Yes
- No
- I do not know

If YES, please outline briefly how this is done or give reference. If possible, please additionally provide practical examples or give reference to practical examples.

### 14. Is retrieved evidence checked for risk of sponsorship / funding bias (i.e. bias related to publication based on the potential to support interests of the study's financial sponsor)

- Yes
- No
- I do not know

If YES, please outline briefly how this is done or give reference. If possible, please additionally provide practical examples or give reference to practical examples.

### 15. Are unpublished or part-published studies assessed using the same validity criteria applied to published studies?

- Yes
- No
- Not applicable (unpublished evidence is not considered at all)
- I do not know

### 16. Is retrieved evidence checked against quality reporting standards (e.g. statements of CONSORT, PRISMA, TREND, STROBE)?

- Yes
- No
- I do not know

### 17. Is retrieved evidence assessed for consistency …

| | Yes | No | I do not know |
|---|---|---|---|
| … within the same document/publication (e.g. methods vs. results section, text vs. tables/figures)? | | | |
| … between different information sources related to same study (e.g. study protocol vs. clinical study report vs. study registry report vs. data submitted by manufacturers vs. published articles)? | | | |

Comments (free text box):

18. **In case of inconsistency of data between various sources of information: Which is the preferred source for synthesis of evidence?**

- Published articles
- Clinical study report
- Study registry report
- Data submitted by manufacturers
- No source is preferred over others
- Not applicable (e.g. single source of information only)
- Other (please specify)

19. **Which of the following methodological approaches related to unpubished or incomplete data are used in quantitative synthesis of results (meta- analysis)? (Please tick all options that apply)**

- Missing data is calculated or estimated, when possible (e.g. effect estimate or variance)
- Uncertainty of synthesis results due to unpublished or incomplete data is explored (i.e. testing for robustness of results as part ofsensitivity analysis or subgroup analysis)
- Individual patient data metaanalysis is used in order to include results of unpublished studies
- Information of ongoing studies incorporated in synthesis of evidence
- Methods are applied to adjust for observed publication bias (e.g. trim and fill technique)
- No approach related to unpubished or incomplete data is used
- Other (please specify)

20. **When interpreting assessment results and concluding or recommending on benefit of pharmaceuticals in the context of reimbursement or pricing: Is unpublished or incomplete data an issue to be considered explicitly?**

- Yes
- No
- I do not know

If YES, please outline briefly how this is done or give reference. If possible, please additionally provide practical examples or give reference to practical examples.

21. **Does your institution accept submissions that include evidence in confidence, i.e. information which is not to be published or only part published during or after the benefit assessment process?**

- Yes
- No
- I do not know

### 22. Which kind of data considered for benefit assessment of pharmaceuticals is made available to … (Please tick all options that apply)

| | ... the public? | ... decision-makers on reimbursement or pricing? | ... stakeholders involved in assessment process? | ... scientific reviewers of evidence? |
|---|---|---|---|---|
| Unpublished data marked as commercial in confidence (i.e. company or business secrets) | | | | |
| Unpublished data marked as academic in confidence (i.e. research secrets) | | | | |
| Unpublished data NOT marked as in confidence | | | | |
| Published data marked as in confidence | | | | |
| Published data | | | | |
| No data | | | | |

Comments (free text box):

### 23. Who is obliged to declare potential conflicts of interest (CoI) during benefit assessment process of pharmaceuticals? (Please tick all options that apply)

- Appointees or staff of assessment commission, committee or institution
- Manufacturers or sponsors
- Consulting patients/consumers or representatives of their associations
- Consulting health professionals or representatives of their associations (physicians, pharmacists, etc.)
- Consulting representatives of hospital associations
- Consulting representatives of payers’ associations
- No one
- Other (please specify)
- 

### 24. How are a person’s relevant CoI handled?

- Person is completely excluded from assessment process
- Person is excluded from voting in assessment process only
- Person is involved in assessment process under exceptional circumstances only
- Person is involved in assessment process without restrictions
- Other (please specify)

## 25. How are CoI made transparent to the public?

- CoI are not published
- Only a summary of a person's CoI is published
- A person's detailed CoI are published
- Other (please specify)

## 26. Which of the following policies are ALREADY applied in your country in order to avoid bias due to unpublished or incomplete data in benefit assessment and decision-making on reimbursement and pricing of pharmaceuticals? (Please tick all options that apply)

- Noncommercial sponsorship or funding of studies
- Study registries as standard source of information for benefit assessments
- Mandatory registration of clinical studies at inception
- Mandatory standardised posting of full study protocols to study registries
- Mandatory standardised posting of full study results to study registries
- Quality standards for study registries
- Public access to study registries
- Public access to national regulatory databases
- Public access to European Medicines Agency's databases
- Public access to study reports prepared for regulatory drug approval
- Public access to studies methods and results of older studies not covered by mandatory study registration
- Data sharing procedures between regulatory authorities and benefit assessment/HTA bodies
- Legal obligation for manufacturers to submit all requested evidence
- Requirement for manufacturers to sign a statement or agreement declaring that all relevant evidence has been submitted
- Financial sanctions for manufacturers not complying to submission requirements
- Nonfinancial sanctions for manufacturers not complying to submission requirements
- None
- Other (please specify)

## 27. Please answer from the viewpoint of your institution: Which of the following policies should be ADDITIONALLY applied in your country in order to avoid bias due to unpublished or incomplete data in benefit assessment and decision-making on reimbursement and pricing of pharmaceuticals? (Please tick all options that apply)

- Noncommercial sponsorship or funding of studies
- Study registries as standard source of information for benefit assessments
- Mandatory registration of clinical studies at inception
- Mandatory standardised posting of full study protocols to study registries
- Mandatory standardised posting of full study results to study registries
- Quality standards for study registries
- Public access to study registries
- Public access to national regulatory databases

- Public access to European Medicines Agency's databases
- Public access to study reports prepared for regulatory drug approval
- Public access to studies methods and results of older studies not covered by mandatory study registration
- Data sharing procedures between regulatory authorities and benefit assessment/HTA bodies
- Legal obligation for manufacturers to submit all requested evidence
- Requirement for manufacturers to sign a statement or agreement declaring that all relevant evidence has been submitted
- Financial sanctions for manufacturers not complying to submission requirements
- Nonfinancial sanctions for manufacturers not complying to submission requirements
- None
- Other (please specify)

### 28. If evidence is incomplete: Which of the following consequences are ALREADY applied in your country during the process of benefit assessment and decision making on reimbursement and pricing of pharmaceuticals? (Please tick all options that apply)

- Benefit assessment can be refused entirely
- Benefit assessment can be postponed until evidence is completed
- Assessment of pharmaceutical(s) can state that benefit is not proven
- Conclusion on benefit of pharmaceutical(s) can be negative
- Recommendation on use of pharmaceutical(s) can be negative
- Decision-making on reimbursement or pricing can generally be refused entirely
- Decision-making on reimbursement or pricing can be postponed until evidence is completed
- (Complete) reimbursement of pharmaceutical(s) can be refused
- (High) pricing of pharmaceutical(s) can be refused
- None
- Other (please specify)

### 29. Please answer from the viewpoint of your institution: If evidence is incomplete: Which consequences should be ADDITIONALLY applied in your country during the process of benefit assessment and decision making on reimbursement and pricing of pharmaceuticals if evidence is incomplete? (Please tick all options that apply)

- Benefit assessment can be refused entirely
- Benefit assessment can be postponed until evidence is completed
- Assessment of pharmaceutical(s) can state that benefit is not proven
- Conclusion on benefit of pharmaceutical(s) can be negative
- Recommendation on use of pharmaceutical(s) can be negative
- Decision-making on reimbursement or pricing can generally be refused entirely
- Decision-making on reimbursement or pricing can be postponed until evidence is completed
- (Complete) reimbursement of pharmaceutical(s) can be refused
- (High) pricing of pharmaceutical(s) can be refused
- None
- Other (please specify)

30. **Do you have any further information on your institution’s policy towards unpublished or incomplete data? Please comment here:**

31. **Do you have any comments on the questionnaire? Please comment here:**

## Part B Review of information and supplementary material

For your country, we drafted a table and chart describing the process of benefit assessment and reimbursement and pricing of pharmaceuticals. We additionally performed a systematic search for regulatory documents on the internet and for literature in bibliographic databases addressing policies of involved institutions towards unpublished or incomplete data in your country. At this stage, we would be grateful if you could add any validating comments on the table and chart and provide us with supplementary material addressing the institutions' policies towards unpublished or incomplete data in your country.

In summary, we kindly ask you

1. to shortly review the table and chart for completeness and correctness and give short answers to a few questions provided in a red box,

2. to provide additional material addressing the institutions' policies towards unpublished or incomplete data in your country (e.g. guideline, directive, code of procedure, law, provision, decree, official statement, statute, declaration, regulation, recommendation, guidance, manual, submission requirements, submission templates, description of methods, etc.).

Please find information and questions concerning your country in the attachment of your invitation email. Please send your comments and supplementary material to dimitra.panteli@tuberlin.de

Thank you for your participation and support!
