# Supplementary material for: Published and unpublished evidence in coverage decision-making for pharmaceuticals in Europe: existing approaches and way forward
Source: Health Res Policy Syst. 2016 Jan 26;14:6. doi: 10.1186/s12961-016-0080-9 (PMC4727332; doi:10.1186/s12961-016-0080-9)
Supplement: Additional file 5: — List of regulatory documents organized per country and list of publications. (PDF 274 kb) [file 12961_2016_80_MOESM5_ESM.pdf]

Additional file 5 List of regulatory documents organized per country and list of publications

Included regulatory documents

Country	Title of regulatory document	Year	Regulatory institution
Austria (AUT)	Handbuch für EBM-Berichte [Handbook for EBM-Reports]	2008	HVB
	Verfahrensordnung zur Herausgabe des Erstattungskodex nach § 351g ASVG-VO-EKO [Code of Procedure for issuing the reimbursement list according to §351g ASVG]Verfahrensordnung zur Herausgabe des Erstattungskodex nach § 351g ASVG - VO-EKO 2004]	2004	HVB
Belgium (BEL)	Commission de Remboursement des Médicaments [Commission on Reimbursement of Pharmaceuticals]	2001	MoSA/CRM
Bulgaria (BGR)	Правила за работа на Комисия по цени и реимбурсиране [Rules of the Committee on PRicing and Reimbursement]	2012	CPR
	Закон за лекарствените продукти в хуманната медицина [Law on Medicinal Products for Human Use]	2011	MoH/CPR
Croatia (HRV)	Pravilnik o mjerilima za stavljanje lijekova na osnovnu i dopunsku listu lijekova hravatskog zavoda za zdravstveno osiguranje [Rulebook on the criteria for inclusion of drugs on the basic and supplementary list of medicines by the Croatian Institute for Health Insurance]	2009	HZZO
Czech Republic (CZR)	N/A		
Denmark (DNK)	Health Technology Assessment Handbook	2007	DACEHTA
Estonia (EST)	Ravimite farmakoökonoomiline hindamine [Pharmacoeconomic evaluation of medicinal products]	n.a.	EHIF
	Eesti Haigekassa ravimite loetelu koostamise ja muutmise kord ning loetelu kehtestamise kriteeriumide sisu ja kriteeriumidele vastavuse hindajad [Procedure and evaluation criteria for establishing Estonian Health Insurance Fund drug list]	2011	EHIF
Finland (FIN)	Decree of the Ministry of Social Affairs and Health on applications and price notifications made to the Pharmaceuticals Pricing Board	2009	MoSAH/PPB
France (FRA)	Règlement intérieur de la Commission de la transparence [Internal Regulation of the Transparency Committee]	2011	HAS
	Guide des déclarations d'intérêts et de gestion des conflits d'intérêts [Guide for the declaration of interests and management of conflicts of interest]	2010	HAS
	Dossier-type pour une demande d'inscription ou de modification des condition d'inscription (notamment extension des indications) sur la liste des médicaments remboursables aux assurés sociaux et/ou sur la liste des médicaments agreés a l'usage des collectives et divers services publics [Specificiations for dossiers for an application for registration or amendment of registration conditions (including indication extension) on the list of medicines reimbursed by National Insurance and / or on the list of authorized medicines for collective use in public services 2007]	2007	HAS

Abbreviations:

CPR – Commission for Pricing and Reimbursement
CRM – Commission de Remboursement des Médicaments
DACEHTA – Danish Centre for Health Technology Assessment
EHIF – Estonian Health Insurance Fund
HAS – Haute Authorité de Santé
HVB – Association of Austrian Social Security Institutions
HZZO – Croatian Institute for Health Insurance
MoH – Ministry of Health
MoSA – Ministry of Social Affairs
MoSAH/PPB – Ministry of Social Affairs and Health/ Pharmaceuticals Pricing Board

Included regulatory documents (cont.)

Country	Title of regulatory document	Year	Regulatory institution
Germany (DEU)	Verfahrensordnung des Gemeinsamen Bundesausschuss [Code of Procedure of the Federal Joint Committee in the version of 18 December 2008, last modified 19 January 2012]	2012	G-BA
	Anlage II.1 zum 5. Kapitel der Verfahrensordnung des Gemeinsamen Bundesausschusses. Erstellung und Einreichung eines Dossiers zur Nutzenbewertung gemäß § 35a SGB V. [Annex II.1 to Chapter 5 of the Code of Procedure of the Federal Joint Committee. Creation and submission of a dossier for benefit assessment following §35a SGB V]	2011	G-BA
	Anlage II.6 zum 5. Kapitel der Verfahrensordnung des Gemeinsamen Bundesausschusses. Modul 4 - Medizinischer Nutzen und medizinischer Zusatznutzen, Patientengruppen mit therapeutisch bedeutsamem Zusatznutzen (Dokumentvorlage, Version vom 20.01.2011) [Annex II.6 to Chapter 5 of the Code of Procedure of the Federal Joint Committee. Module 4 - medical benefit and medical additional benefit, patient groups with medically meaningful additional benefit]	2011	G-BA
	Allgemeine Methoden Version 4.0 [General Methods Version 4.0]	2011	IQWiG
Greece (GRC)	N/A		
Hungary (HUN)	N/A		
Iceland (ISL)	N/A		
Ireland (IRL)	Guidelines for Evaluating the Clinical Effectiveness of Health Technologies in Ireland	2011	HIQA
	Guidelines for the Economic Evaluation of Health Technologies in Ireland	2010	HIQA
Italy (ITA)	N/A		
Latvia (LVA)	N/A		
Lithuania (LTU)	N/A		
Luxembourg (LUX)	Règlement grand-ducal modifié du 12 décembre 2002 précisant les conditions et déterminant la procédure relatives à l'inscription d'un médicament sur la liste positive des médicaments pris en charge par l'assurance maladie. [Grand Ducal Regulation as amended on 12 December 2002 detailing the conditions and determining the procedure for the registration of a drug on the positive list of medicines covered by health insurance]	2002	UCM

Abbreviations:

G-BA – Gemeinsamer Bundesausschuss (Federal Joint Committee)
IQWIG – Institut für Qualität und Wirtschaftlichkeit im Gesundheitswesen (Institute for Quality and Efficiency in Health Care)
HIQA – Health information and Quality Authority
UCM – Union of Sickness Funds

Included regulatory documents (cont.)

Country	Title of regulatory document	Year	Regulatory institution
Malta (MLT)	N/A		
Macedonia (MKD)	N/A		
Montenegro (MNE)	N/A		
The Netherlands (NLD)	Procedure beoordeling extramurale geneesmiddelen [Procedure for assessing outpatient drugs]	2011	CVZ
	Procedure beoordeling intramurale geneesmiddelen [Procedure for assessing inpatient drugs]	2006	CVZ
	Guidelines for pharmacoeconomic research	2006	CVZ
Poland (POL)	Guidelines for conducting Health Technology Assessment (HTA)	2009	AOTM
Portugal (PRT)	N/A		
Romania (ROU)	N/A		
Serbia (SRB)			
Slovakia (SVK)	Metodická pomôcka pre vykonávanie farmako-ekonomického rozboru lieku, medicínsko-ekonomického rozboru zdravotníckej pomôcky a medicínsko-ekonomického rozboru dietetickej potraviny [Guidelines for implementation of pharmaco-economic analysis of medicines, medico-economic analysis of medical devices and dietary food]	2008	MoH
Slovenia (SVN)	N/A		
Spain (ESP)	N/A		
Sweden (SWE)	Handbok för företag vid ansökan om subvention och pris för läkemedel [Practice handbook for applications for reimbursement and pricing]	2012	TLV
	Tandvårds- och läkemedelsförmånsverkets föreskrifter och allmänna råd om ansökan och beslut om läkemedel och varor som förskrivs i födelsekontrollerande syfte [Regulations and general guidelines of the Dental and Pharmaceutical Benefits Agency for the application for and decision-makinhg about drugs and products that are prescribed in birth control]	2008	TLV
	Act (2002:160) on Pharmaceutical Benefits, etc.	2002	TLV

Abbreviations

CVZ - College voor Zorgverzekeringen (Medicinal Products Reimbursement Committee of the Dutch Healthcare Insurance Board, now National Health Care Institute)
AOTM - Agency for Health Technology Assessment in Poland
MoH – Ministry of Health
TLV - Dental and Pharmaceutical Benefits Agency

Included regulatory documents (cont.)

Country	Title of regulatory document	Year	Regulatory institution
Turkey (TUR)	Ödeme komisyonunun çalışma usul ve esasları hakkındaki yönerge [Directive on working procedures and principles of the reimbursement commission]	2012	SGK
UK - England & Wales (ENG)	Specification for manufacturer/sponsor submission of evidence.	2012	NICE
	Agreement between the Association of the British Pharmaceutical Industry (ABPI) and the National Institute for Clinical Excellence (NICE) on guidelines for the release of company data into the public domain during a health technology appraisal.	2011	NICE
	Guide to the Multiple Technology Appraisal Process (reference N2022).	2009	NICE
	Guide to the single technology appraisal process	2009	NICE
	Guide to the methods of technology appraisal.	2008	NICE
	A Code of practice for Declaring and Dealing with Conflicts of Interest. last amended October 2008	2008	NICE
	Contributing to a Technology Appraisal: A Guide for Healthcare Professional Groups (reference N0517)	2004	NICE
	Contributing to a Technology Appraisal: A Guide for Manufacturers and Sponsors (reference N0518)	2004	NICE
	Contributing to a Technology Appraisal: A Guide for NHS Organisations (reference N0519)	2004	NICE
	Appraisal Process: Guidance for Appellants (reference N0520)	2004	NICE
UK – Scotland (SCT)	Guidance to Manufacturers for Completion of New Product Assessment Form (NPAF)	2012	SMC
Switzerland (CHE)	Allgemeine Bestimmungen zur Spezialitätenliste	2011	BAG/ EAK
	Geschäftsordnung	2009	BAG
Liechtenstein (LIE)	N/A		
Norway (NOR)	Application standard for acceptance to the drug reimbursement scheme; pursuant to Article 9 of the regulation on reimbursement of crucial drug costs	2005	SLV
	Norwegian Guidelines on how to conduct pharmacoeconomic analyses	2012	SLV

Abbreviations:

SGK – Social Security Institution
NICE – National Institute for Health and Care Excellence
SMC – Scottish Medicines Consortium

BAG – Federal Office of Public Health
EAK – Eidgenössische Arzneimittelkommission (Federal Drug Commission)
SLV – Statens Legemiddelverk (Norwegian Medicines Agency)

Included publications

Title of publication	Year	Author/s	Country	Regulatory institution
Conflicts of interests of experts in the field of health: The decision of the "conseil d'Etat" for the annulment of a recommendation of the "haute Autorite de sante". [French]	2012	Duguet AM	France	HAS
Impact of document type on reporting quality of clinical drug trials: a comparison of registry reports, clinical study reports, and journal publications	2012	Wieseler B et al.	Germany	IQWiG
Finding studies on reboxetine: a tale of hide and seek.	2010	Wieseler B, McGauran N, Kaiser T	Germany	IQWiG
Reporting bias in medical research - a narrative review.	2010	McGauran N et al.	Germany	IQWiG
Results registries for clinical trials-a milestone on the way to transparency in clinical research? [German].	2010	Wieseler B	Germany	IQWiG
Searching clinical trials registries: procedure and documentation. [German]	2010	Hausner E and Kaiser T	Germany	IQWiG
German agency refuses to rule on drug's benefits until Pfizer discloses all trial results.	2009	Stafford N	Germany	IQWiG
Rationing new medicines in the UK	2009	Drummond M	England & Wales	NICE
Sequestered evidence and the distortion of clinical practice guidelines.	2009	McGoey L	England & Wales	NICE
Truth, Disclosure and the Influence of Industry on the Development of NICE Guidelines: An Interview with Tim Kendall	2007	Kendall T and McGoey Linsey	England & Wales	NICE
Identification and assessment of ongoing trials in health technology assessment reviews.	2004	Song FJ et al.	England & Wales	NICE
Literature searching for clinical and cost-effectiveness studies used in health technology assessment reports carried out for the National Institute for Clinical Excellence appraisal system.	2003	Royle P and Waugh N	England & Wales	NICE
Clinical effectiveness and cost effectiveness of zanamivir (Relenza): translating the evidence into clinical practice, a National Institute for Clinical Excellence view.	2001	Barnett D	England & Wales	NICE

Abbreviations:

HAS – Haute Authorité de Santé

IQWiG – Institut für Qualität und Wirtschaftlichkeit im Gesundheitswesen (Institute for Quality and Efficiency in Health Care)

NICE – National Institute for Health and Care Excellence
