# Supplementary material for: Published and unpublished evidence in coverage decision-making for pharmaceuticals in Europe: existing approaches and way forward
Source: Health Res Policy Syst. 2016 Jan 26;14:6. doi: 10.1186/s12961-016-0080-9 (PMC4727332; doi:10.1186/s12961-016-0080-9)
Supplement: Additional file 6: Table S3. — Sources used to retrieve information for the assessment of pharmaceuticals among respondents to the survey (n = 13). (DOCX 16 kb) [file 12961_2016_80_MOESM6_ESM.docx]

Table 3 Sources used to retrieve information for the assessment of pharmaceuticals among respondents to the survey (n = 13)

| Answer Options | always | occasionally | never |
| --- | --- | --- | --- |
| Published scientific literature | 11 | 2 | 0 |
| European Medicines Agency (EMA) | 9 | 4 | 0 |
| National regulatory agencies for marketing authorisation | 8 | 5 | 0 |
| Manufacturers or sponsors | 7 | 6 | 0 |
| Clinical guidelines | 7 | 6 | 0 |
| Other public bodies in home country (e.g. ministry, committees) | 6 | 5 | 2 |
| Other coverage/HTA institutions in home country | 5 | 6 | 2 |
| Food and Drug Administration (FDA) | 4 | 9 | 0 |
| Coverage/HTA institutions in other European countries | 4 | 7 | 2 |
| Halthcare professionals or their associations (e.g. physicians, pharmacists) | 4 | 9 | 0 |
| Study authors, researchers, or investigators | 2 | 9 | 2 |
| European or international networks of assessment bodies/HTA institutions  (e.g. HEN, INAHTA, EUnetHTA, etc.) | 2 | 8 | 3 |
| Hospitals or their associations | 2 | 8 | 3 |
| Payers or their associations | 2 | 6 | 5 |
| Clinical trial (meta-)registries | 1 | 10 | 2 |
| Disease registries | 1 | 10 | 2 |
| Coverage/HTA institutions outside Europe | 1 | 10 | 2 |
| Patients/consumers or their associations | 1 | 6 | 6 |

Note: numbers correspond to absolute number of responses
