# Supplementary material for: Published and unpublished evidence in coverage decision-making for pharmaceuticals in Europe: existing approaches and way forward
Source: Health Res Policy Syst. 2016 Jan 26;14:6. doi: 10.1186/s12961-016-0080-9 (PMC4727332; doi:10.1186/s12961-016-0080-9)
Supplement: Additional file 7: Table S4. — Bibliographic databases used to identify published information among respondents (n = 13). (DOCX 15 kb) [file 12961_2016_80_MOESM7_ESM.docx]

Table 4 Bibliographic databases used to identify published information among respondents (n = 13)

| Database | Absolute number of responses |
| --- | --- |
| Medline | 10 |
| Cochrane Library | 9 |
| Embase | 4 |
| CRD Databases (DARE, NHS-EED, HTA-Database) | 3 |
| CINAHL | 2 |
| PsychINFO | 2 |
| TRIP-Database | 1 |
| Science Citation Index | 1 |
| Other | 3 |

Note: Multiple responses were possible
